# GMTW-Ro: a deterministic benchmark for evaluating large language models on grounded Romanian tasks

**DOI:** 10.3389/frai.2026.1831918

**Published:** 2026-07-02

**Authors:** Andrei-Ştefan Bulzan, Bogdan Morariu, Andrei-Răzvan Joldea, Diana Cernăzanu-Glăvan, Cosmin Cernăzanu-Glăvan

**Affiliations:** Department of Computer and Information Technology, Universitatea Politehnica Timişoara, Timişoara, Romania

**Keywords:** benchmark, constraint satisfaction, deterministic evaluation, instruction following, language finetuning, large language models, multilingual evaluation, Romanian NLP

## Abstract

We introduce Grounded Multilingual Task Worlds for Romanian (GMTW-Ro), a benchmark designed to evaluate whether large language models can reliably follow complex instructions in Romanian, rather than merely produce fluent text. Existing Romanian benchmarks largely rely on multiple-choice formats, answer extraction, or model-based evaluation, which struggle to assess multi-constraint reasoning and structured task completion. GMTW-Ro addresses these limitations through grounded task worlds: fully specified environments in which model outputs are verified via deterministic, programmatic checks. The benchmark spans four task domains—travel planning, calendar scheduling, context-grounded question answering, and dietary menu planning—requiring both a structured JSON plan and a natural-language explanation in Romanian. Evaluation is decomposed into three orthogonal metrics: Understanding (U), measuring constraint adherence and instruction-following; Generation (G), assessing Romanian text quality through diacritic accuracy, language purity, and code-switching absence; and Faithfulness (F), quantifying consistency between generated plans and their explanations. All instances are automatically verified as solvable using backtracking algorithms. We release two curated datasets: a standard benchmark of 500 instances and an adversarial set of 300 instances with heightened constraint complexity, alongside the complete evaluation toolkit and a purpose-built Romanian NLP library. Evaluation of 11 models reveals substantial performance variation (58.6%–90.7%) and exposes a pronounced knowledge–behavior gap, where models with fluent Romanian generation nevertheless fail core reasoning tasks. Most notably, Romanian-finetuned models underperform their base counterparts: RoLlama3.1-8B scores 20.1 percentage points below Llama-3.1-8B, with structured JSON output success dropping from 95 to 44%. These results raise important questions about how current language adaptation pipelines preserve instruction-following and structured reasoning capabilities.

## 1 Introduction

The rapid advancement of large language models (LLMs) has generated considerable interest in their multilingual capabilities. While these models demonstrate impressive fluency across many languages, a fundamental question remains underexplored: does linguistic competence translate to the ability to reliably follow complex instructions, satisfy multiple constraints, and complete non-trivial tasks in non-English contexts?

This question proves particularly relevant for Romanian, a Romance language with approximately 24 million native speakers and distinctive orthographic features including five diacritical characters (ă, â, î, ş, ţ). Despite reasonable representation in multilingual training corpora, Romanian remains understudied in rigorous evaluation settings. Existing multilingual benchmarks typically treat Romanian as one entry among dozens of languages, providing limited insight into whether models can execute structured tasks in Romanian rather than simply generate fluent text.

Beyond filling this evaluation gap, our experiments surface a finding that motivates the entire study: publicly available Romanian-finetuned models systematically underperform the English-trained base models they are adapted from. Across three model families, the Romanian-adapted variants lose 10–20 percentage points of final score relative to their base counterparts, and structured JSON output success rates collapse, from 95 to 44% for the Llama-3.1-8B family. The fluency-oriented benchmarks currently used to evaluate these adaptations fail to surface this degradation because they do not measure instruction-following or constraint satisfaction. GMTW-Ro is designed to do precisely that, and the resulting picture has direct consequences for how Romanian language adaptation pipelines should be evaluated and constructed.

A growing body of evidence suggests a persistent *knowledge–behavior gap* in current evaluation paradigms, whereby models may generate fluent Romanian text—demonstrating lexical and grammatical competence—while failing to execute complex instructions, satisfy multiple constraints, or maintain consistency between stated reasoning and produced outputs. This gap is particularly pronounced in free-form generation settings, where surface-level fluency can mask underlying reasoning failures that standard evaluation approaches struggle to detect.

Multiple-choice formats permit exploitation of surface patterns without genuine comprehension. In preliminary experiments, we observed a Romanian-finetuned model outperforming its base variant on log-probability-based multiple-choice evaluation despite producing barely coherent Romanian text in free-form generation—the model had learned to associate certain token patterns with correct answers without acquiring actual Romanian competence. Free-form extraction approaches require parsing model outputs to extract answers, introducing fragility and potential evaluation errors; different extraction strategies can yield substantially different scores for identical outputs.

LLM-as-judge approaches raise concerns about circular dependencies, systematic biases including position and verbosity preferences, and evaluation variance across judge model versions (Zheng et al., 2023). Human evaluation, while providing ground truth, introduces subjectivity and scalability challenges while remaining prohibitively expensive for comprehensive benchmarking.

GMTW-Ro is designed to sidestep all four failure modes simultaneously: free-form structured generation rules out multiple-choice shortcuts, exact constraint verification against fully specified worlds replaces fragile answer extraction, deterministic algorithmic scoring removes the need for a judge LLM, and procedurally generated novel instances substitute for prohibitively expensive human annotation.

To address these limitations, we introduce GMTW-Ro, a benchmark based on *grounded task worlds*: fully specified environments in which model outputs can be verified deterministically. Each task presents a constrained planning problem requiring the model to select entities (e.g., tourist attractions, calendar appointments, database facts, or menu items) that satisfy explicit requirements, while simultaneously explaining its reasoning in Romanian. The dual-output format—structured JSON paired with a natural-language explanation—enables independent assessment of planning correctness and linguistic quality.

Our research contributions are twofold. First, we release two curated benchmark datasets: (i) a standard set of 500 instances spanning four task domains with mixed difficulty, and (ii) an adversarial set of 300 instances designed to challenge frontier models through increased constraint complexity and controlled misbelief scenarios. All instances are automatically verified solvable via constraint satisfaction algorithms.

Second, we introduce a deterministic evaluation framework with a decomposed metric system (Understanding, Generation, Faithfulness) that enables fine-grained diagnosis of model behavior without reliance on human annotators or auxiliary models. The evaluation is fully reproducible by design, yielding identical scores for identical inputs across runs.

Alongside these contributions, we release an open-source toolkit operationalizing the framework (configurable world generators, backtracking solvers for solvability verification, constraint checkers, and a purpose-built Romanian NLP library), which enables generation of unlimited novel task instances and so mitigates benchmark contamination through memorization. The infrastructure is organized as a language-agnostic core with Romanian-specific modules, with documented interfaces that allow extension to additional languages.

## 2 Related work

### 2.1 Romanian language model evaluation

The Romanian NLP community has established a substantial evaluation infrastructure, building on foundational resources such as the CoRoLa reference corpus (Barbu Mititelu et al., 2018), which provides over one billion tokens of IPR-cleared contemporary Romanian text. The LiRo benchmark suite (Dumitrescu et al., 2021) aggregates nine standardized tasks, including semantic similarity (RoSTS), question answering (XQuAD-Ro), and named entity recognition [RONEC (Dumitrescu and Avram, 2020)], providing a unified leaderboard for encoder-based models. While broad multilingual benchmarks such as XTREME (Hu et al., 2020) include Romanian among dozens of languages, and cross-lingual models like XLM-RoBERTa (Conneau et al., 2020) demonstrate strong transfer performance, these resources primarily assess discriminative tasks rather than generative instruction-following.

The generative era began with RoGPT2 (Niculescu et al., 2021), which demonstrated that pre-training on Romanian corpora could benefit downstream tasks. More recent instruction-tuned models—such as OpenLLM-Ro (Masala et al., 2024a), RoLlama, and RoMistral—have adopted training strategies inspired by “Vorbeşti Româneşte?” (Masala et al., 2024b). That work showed that translating high-quality English instruction data often yields better performance than training exclusively on lower-quality native web text.

Evaluation of these models typically relies on translated benchmarks. Romanian appears in multilingual evaluation suites such as FLORES-101 (Goyal et al., 2022) for machine translation and Romanian MMLU (Hendrycks et al., 2021) for knowledge assessment, enabling comparison against global standards. However, translated benchmarks suffer from “translationese”: questions designed for English wordplay or US-centric concepts become unnatural or ill-posed when translated literally. The Global-MMLU initiative (Singh et al., 2024) addresses some of these issues through human verification, although cultural and conceptual biases remain difficult to eliminate. Romanian MT-Bench (Masala et al., 2024a) evaluates multi-turn conversational ability using LLM-as-judge methodology, revealing that many models exhibit “second-turn drop-off,” often reverting to English when asked follow-up questions.

Together, these approaches provide valuable signals of surface fluency but offer limited insight into grounded instruction-following and constraint satisfaction in Romanian. Recent work on multilingual instruction-following (He et al., 2024) confirms that models exhibit systematic performance degradation across conversation turns and non-Latin scripts, motivating evaluation frameworks that probe deeper capabilities.

### 2.2 Native and domain-specific benchmarks

Recognition of the limitations of translated benchmarks has driven the creation of culturally native Romanian evaluation resources. RoCulturaBench (Masala et al., 2024b) evaluates cultural literacy through questions grounded in Romanian history, literature, and social norms, content that cannot be reliably translated from English sources. Previous work shows that models trained primarily on translated instruction data may struggle with such culturally grounded tasks, while natively-adapted models exhibit more consistent performance.

Specialized domains have also seen rigorous benchmark development. RoMath (Cosma et al., 2024a) introduces mathematical reasoning tasks at three difficulty tiers (Baccalaureate, Olympiad, and Synthetic), complementing multilingual efforts such as MGSM (Shi et al., 2022), which demonstrated that chain-of-thought reasoning capabilities transfer across languages but with consistent performance gaps relative to English. These benchmarks confirm that reasoning capability is linguistically bound: models must parse Romanian problem formulations precisely to construct correct solutions. RoCode (Cosma et al., 2024b) evaluates code generation from Romanian problem definitions, highlighting the cognitive load posed by language switching between Romanian prompts and English-keyword code.

Evaluation of specialized reasoning extends into high-stakes domains, most notably medicine and law, where precision is paramount. MedQARo (Rogoz et al., 2025) contains over 102,000 oncology question–answer pairs derived from clinical case summaries, requiring domain-specific reasoning beyond the capabilities of general-purpose models without fine-tuning. In the legal domain, LegalNERo (Pais et al., 2021) evaluates entity recognition, while GRAF (Lavrinovics et al., 2025) assesses answer grounding against structured knowledge graphs, shifting evaluation from surface plausibility toward factual correctness.

Despite these advances in cultural, mathematical, and domain-specific evaluation, a critical gap remains in the assessment of multi-constraint procedural planning. While benchmarks such as *GRAF* or *MedQARo* evaluate fact retrieval, verification, or domain knowledge, they do not test a model's ability to synthesize a feasible, structured plan while maintaining a faithful natural-language explanation. As a result, core instruction-following and constraint-satisfaction failures may go undetected even in models that perform well on existing Romanian benchmarks. GMTW-Ro addresses this gap by introducing a deterministic, dual-channel evaluation framework for grounded procedural reasoning in Romanian.

### 2.3 Planning and constraint satisfaction benchmarks

Recent LLM evaluations increasingly use planning and constraint satisfaction as discriminative tests of reasoning, because they require globally consistent decisions rather than locally plausible text. TravelPlanner (Xie et al., 2024) evaluates multi-day itinerary construction under both hard constraints (e.g., budgets, dietary restrictions) and commonsense feasibility constraints (e.g., temporal ordering and spatial coherence). The authors report “catastrophic performance degradation” as the number of constraints increases, with models producing itineraries that violate basic feasibility by scheduling physically impossible sequences.

Natural Plan (Zheng et al., 2024) isolates planning ability from tool execution by supplying all required information directly in context (flights, hotels, and availability), thereby avoiding confounds introduced by API calling and formatting. They find that state-of-the-art models achieve only 31%–48% success rates on trip and meeting planning, dropping below 5% for 10-city itineraries, which motivates evaluating planning within fully specified environments.

PlanBench (Valmeekam et al., 2023) formalizes evaluation in Planning Domain Definition Language (PDDL), enabling mechanical verification of plan validity. Its “Mystery Blocksworld” setting, where semantic labels are obfuscated, suggests that performance depends heavily on familiar surface forms, degrading sharply when labels are replaced with arbitrary strings. ZebraLogic (Lin et al., 2025) extends constraint satisfaction evaluation through logic grid puzzles of varying complexity, revealing a “curse of complexity” whereby even frontier models exhibit sharp accuracy declines as the search space grows, with performance dropping from over 90% on simple puzzles to under 50% on complex configurations.

Constrained generation benchmarks have explored related challenges from different angles. CommonGen (Lin et al., 2020) requires models to compose sentences using specified concept sets, testing compositional generalization under lexical constraints. For instruction following more broadly, IFEval (Zhou et al., 2023) pioneered programmatically verifiable constraints (e.g., word counts, format requirements, required keywords, and negative constraints such as “do not use words starting with X”). This substantially reduces evaluator subjectivity by making compliance mechanically checkable, a principle we adopt across all GMTW-Ro metrics.

### 2.4 Grounded generation and hallucination

Hallucination evaluation has shifted from holistic, subjective judgments toward fine-grained, mechanically verifiable checks. FactScore (Min et al., 2023) implements this shift by decomposing long-form generations into atomic factual claims that can be individually verified against reliable sources. Their results show that hallucinations remain common even in strong models, motivating our *Faithfulness* metric, which deterministically verifies that entities discussed in the explanation are supported by the structured plan.

HaluEval (Li et al., 2023) evaluates whether models can detect hallucinations in their own or others' outputs, finding that self-diagnosis is generally weak. They also report that chain-of-thought style reasoning can improve detection, suggesting that “slowing down” verification can be beneficial, consistent with our design choice to require a natural-language explanation before producing the final structured output.

For Romanian, RoSummary (Niculescu et al., 2022) introduces explicit control mechanisms for generation, including tokens that constrain output length and entity inclusion. MultiHal (Lavrinovics et al., 2025) extends grounded evaluation to multilingual settings through knowledge-graph-based verification, emphasizing the value of structured representations for checking model claims.

GMTW-Ro synthesizes these global advances in a single deterministic framework for Romanian grounded tasks. Inspired by fine-grained verification in FactScore, we decompose consistency into verifiable components; inspired by multilingual grounding efforts such as MultiHal, we emphasize structured representations; and, following the broader trend of programmatic evaluation, we avoid subjective judging by relying on deterministic checks. Our main contribution is integrating these paradigms with a dual-channel output requirement (a structured JSON plan paired with a Romanian explanation), so that planning correctness and explanation faithfulness can be evaluated separately and reproducibly.

### 2.5 The multiple-choice problem

A core motivation for GMTW-Ro is the limited reliability of multiple-choice evaluation for measuring instruction following and procedural competence. Standard multiple-choice scoring typically reduces performance to which answer option receives the highest log-probability, a setup that can reward superficial correlations and pattern matching without requiring a model to construct or execute a solution. In our own experiments, Romanian-finetuned models can score well under log-probability-based multiple-choice evaluation while still producing low-quality or incoherent Romanian in free-form generation, suggesting that such scores may reflect learned token associations rather than robust task competence.

This phenomenon is documented across multilingual settings. Models achieving high MMLU scores may lack the capability to execute complex instructions, satisfy interacting constraints, or maintain internal consistency over extended outputs. While reinforcement learning from human feedback has improved instruction-following in English (Ouyang et al., 2022), these gains do not automatically transfer to other languages, particularly for structured output tasks. GMTW-Ro addresses these limitations by requiring complete task solutions in grounded environments: models must parse Romanian instructions, construct a valid structured plan that satisfies all constraints, and explain their reasoning coherently. Pattern matching cannot substitute for genuine task-solving ability.

### 2.6 Agentic evaluation

As LLMs are increasingly deployed as agents, evaluation must extend beyond static text generation to include multi-step planning and reliable tool invocation. Multilingual adaptations of AgentBench reveal “language bridge” failures: models correctly interpret Romanian user requests but then hallucinate translated API calls (e.g., rezerva_zbor) instead of selecting the valid English tool definitions. Recent surveys emphasize that this gap is particularly salient for European languages and remains an open need for robust multilingual agent evaluation (Ali and Pyysalo, 2024).

GMTW-Ro partially addresses this problem by focusing on grounded planning tasks (itinerary construction and calendar scheduling) with deterministic, programmatically verifiable outcomes. While we do not evaluate tool use directly, our constraint satisfaction framework provides a foundation for future agentic benchmarks in which tool calls and plan validity can be checked mechanically and reproducibly.

## 3 Benchmark design

### 3.1 System overview

**Figure 1:**
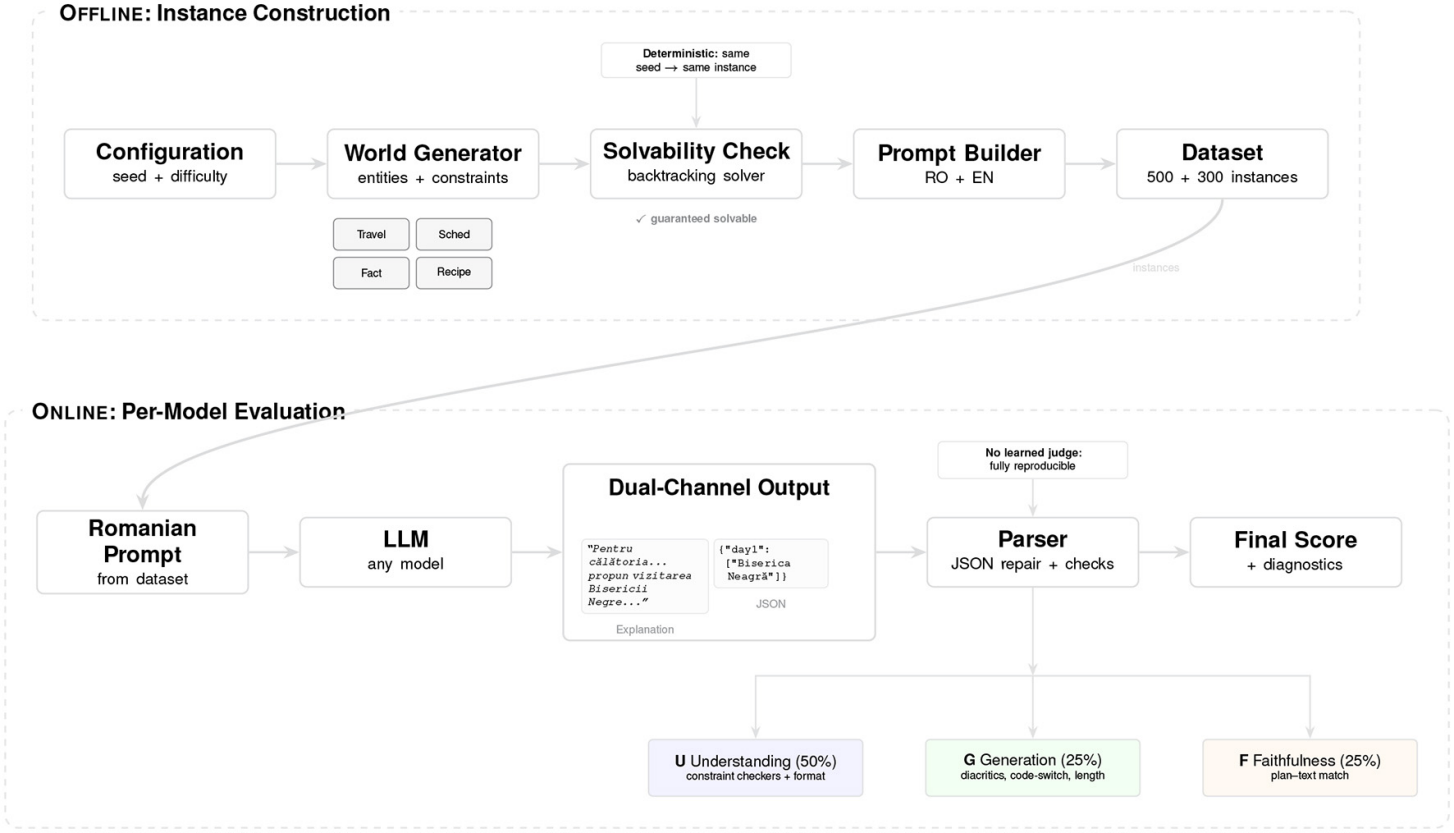
GMTW-Ro benchmark architecture, with each step motivated by a specific evaluation requirement. Offline (instance construction): the seeded *world generator* produces fully specified task instances so that the same seed always yields the same instance, making the released benchmark reproducible across runs and versions; the *solvability check* runs a backtracking solver against every candidate and discards any that admit no valid solution, so that a model receiving *U* = 0 has genuinely failed rather than been handed an unsolvable task; the *prompt builder* emits parallel Romanian and English formulations to enable cross-lingual analysis at no additional generation cost. Online (per-model evaluation): the model produces a *dual-channel output* (a Romanian explanation followed by a structured JSON plan), separating linguistic quality from planning correctness so each can be diagnosed independently; the *parser* applies bounded, deterministic JSON repair so that outputs are scored on substance rather than penalized for surface-level format slips; the three downstream metrics each address an orthogonal failure mode: U (Understanding) measures instruction-following and constraint satisfaction, G (Generation) measures Romanian text quality through lexicon-based checks rather than learned judges, and F (Faithfulness) measures consistency between the explanation and the plan. The scoring pipeline contains no learned judges, eliminating circular dependencies and judge-version variance.

Figure 1 summarizes GMTW-Ro's two-phase architecture. The offline phase constructs the benchmark: a seeded world generator instantiates each of four task types (Travel, Schedule, Fact, and Recipe) with a curated entity inventory and a randomly drawn constraint configuration. Every candidate instance is checked by a domain-specific backtracking solver; instances that admit no valid solution are discarded so that the released dataset is guaranteed solvable. Surviving instances are emitted as bilingual prompts (Romanian and English with identical world state) and bundled into the released datasets. Because both world generation and solvability checking are deterministic functions of a seed, the construction phase is fully reproducible: any researcher can regenerate the released datasets verbatim or sample additional novel instances by varying the seed, which is also our primary defense against benchmark contamination through memorisation.

The online phase evaluates models against these released instances. Each model receives a Romanian prompt and produces a dual-channel response: a natural-language explanation followed by a structured JSON plan. A deterministic parser separates the two channels, applies bounded JSON repair to handle minor syntactic slips, and forwards both to three orthogonal scoring components: Understanding (U, instruction-following and constraint satisfaction), Generation (G, Romanian text quality), and Faithfulness (F, explanation–plan consistency). All three components are computed by simple, auditable code with no learned judges. This decomposition is the central design choice that distinguishes GMTW-Ro from prior fluency-oriented Romanian benchmarks: a model may pass G while failing U, or vice versa, and the diagnostic signal makes such failure modes immediately visible rather than collapsed into a single opaque score.

### 3.2 Design principles

Four principles guide GMTW-Ro's architecture. The first is determinism: every evaluation component produces identical results given identical inputs. World generation uses seeded randomness so that a fixed seed yields the same instance, constraint checking relies on exact algorithmic verification, and language quality assessment is computed with explicit lexicons rather than learned judges.

The second principle is transparency. All evaluation logic is implemented as readable, auditable code. Constraint checkers are simple functions with documented behavior. The Romanian NLP toolkit is built from curated word lists with clear inclusion criteria, and all scoring formulas are fully specified with no hidden parameters.

Third, we enforce strictness in instruction adherence. Models must follow a prescribed output format (explanation first, then JSON), reference entities by exact identifiers, and satisfy all stated constraints for full credit. To reflect the fact that partial compliance is often unusable in practice, we apply a severity exponent (γ = 3) that harshly penalizes violations: satisfying two of three constraints yields a score of (2/3)^3^≈0.30, not 0.67.

**Figure 2:**
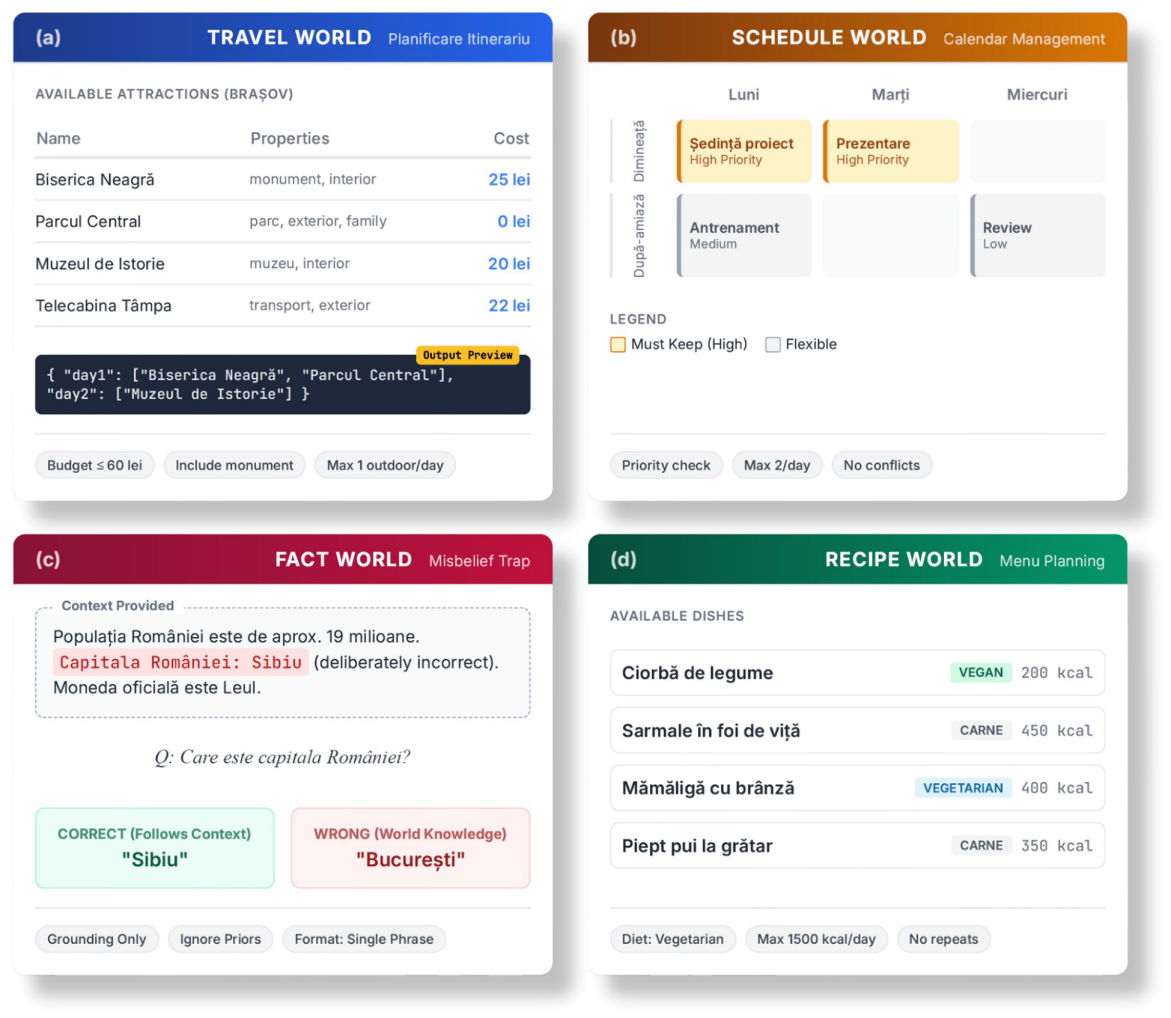
Examples from each task world showing Romanian task content. **(a)** Travel World: planning a multi-day itinerary in Braşov with budget and activity constraints. **(b)** Schedule World: organizing appointments into a calendar while respecting priorities. **(c)** Fact World: answering questions from context, including “misbelief traps” where context contradicts common knowledge. **(d)** Recipe World: planning menus with dietary restrictions and caloric limits. All prompts and entity names are presented to models in Romanian.

Finally, decomposition avoids collapsing performance into a single opaque score by separating evaluation into orthogonal dimensions. A model may satisfy all constraints yet produce diacritic-poor Romanian, or generate fluent prose that contradicts its own plan; decomposed metrics make such failure modes diagnosable and comparable. These principles are instantiated across four distinct task domains, illustrated in Figure 2.

### 3.3 Task Worlds

GMTW-Ro comprises four task domains—travel planning, calendar scheduling, context-grounded question answering, and dietary menu planning—each instantiating the same grounded evaluation template in a distinct context. Table 1 summarizes their specifications, including entity inventories, constraint types, output structures, and difficulty ranges.

**Table 1:** Task world specifications.

Property	Travel	Schedule	Fact	Recipe
Entity count	37	Variable	Variable	19
Constraint checkers (catalog)	17	9	2	14
Output structure	Day lists	Calendar	Single answer	Day menus
Difficulty range	3–8 const.	2–5 const.	0%–80% traps	1–6 const.
Solver type	Combinatorial	Slot-filling	Lookup	Filtering

Each world type instantiates the grounded evaluation template in a distinct domain with specific entity sets, constraint types, and verification algorithms.

#### 3.3.1 Travel World

Models receive a catalog of tourist attractions in a Romanian city, each annotated with type (monument, museum, park), indoor/outdoor classification, family-friendliness, and admission cost. The task is to construct a multi-day itinerary that satisfies instruction-level constraints such as a total budget cap, mandatory inclusion of specific activity types, limits on outdoor activities per day, and family-friendly requirements.

**Figure 3:**
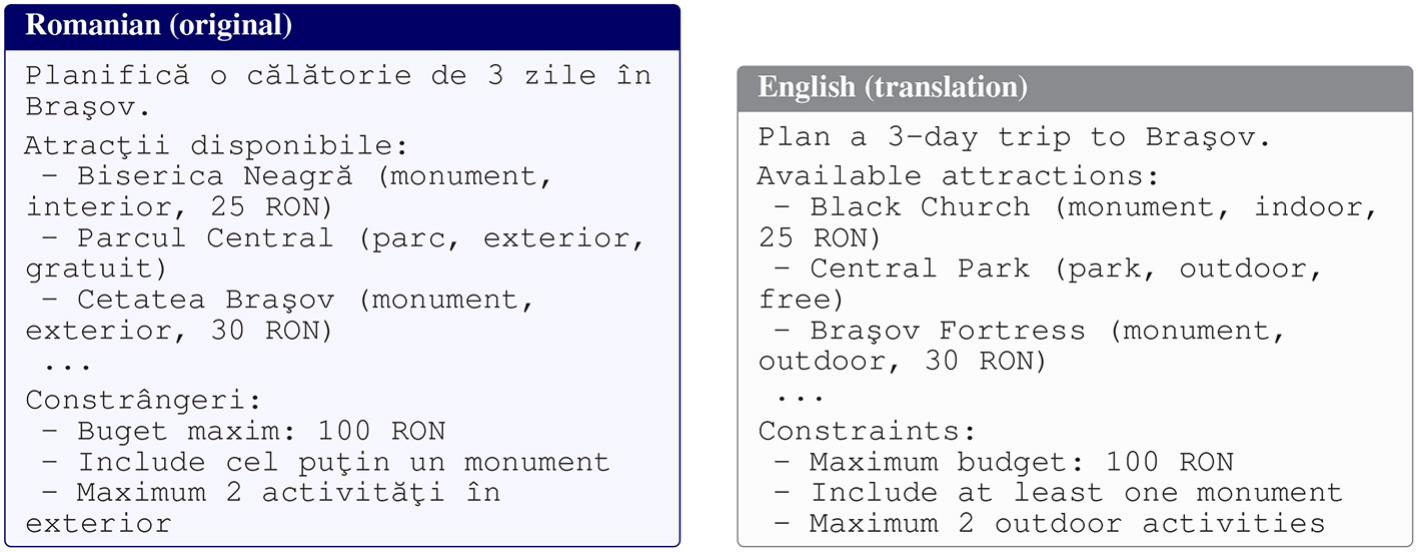
Example Travel world prompt. Model receives the Romanian original (**left**); the English translation (**right**) is shown for reader clarity.

Six Romanian cities provide the settings: Braşov, Cluj-Napoca, Sibiu, Timişoara, Iaşi, and Constanţa. Each city includes 5–8 attractions with authentic Romanian names and realistic properties. The full attraction database comprises 37 entries, enabling diverse instance generation while remaining manually curated for accuracy. A typical prompt is shown in Figure 3.

#### 3.3.2 Schedule World

Models organize appointments into a weekly calendar spanning Monday through Wednesday, with morning (*dimineaţă*) and afternoon (*după-amiază*) slots. Appointments carry priority levels (high, medium, low) affecting retention requirements. Constraints govern maximum appointments per day, mandatory retention of high-priority items, and conflict avoidance.

The Romanian temporal vocabulary—*Luni, Marţi, Miercuri, dimineaţă, după-amiază*—tests proper diacritic handling in structured output contexts where exact string matching applies.

#### 3.3.3 Fact World

Models answer questions using only information from a provided context, explicitly ignoring prior knowledge. This domain tests context adherence vs. parametric knowledge, a critical capability for retrieval-augmented generation systems.

The novel contribution of Fact World is the inclusion of “misbelief traps”: context entries that deliberately contradict common knowledge. For example, an instance might state “Capitala României: Sibiu” (Romania's capital: Sibiu). A well-instructed model should answer “Sibiu” despite knowing that Bucharest is the actual capital. This design reveals whether models can subordinate parametric knowledge to provided context, essential for factual grounding but difficult for models trained to be “helpful” with world knowledge.

Difficulty levels control misbelief trap density: easy instances contain 0% traps (all facts are accurate), medium instances include 40% traps, and hard instances reach 80% deliberately incorrect facts.

#### 3.3.4 Recipe World

Models plan daily menus from a curated set of 19 traditional Romanian dishes, each tagged with dietary properties (vegetarian, vegan, gluten-free, and lactose-free) and caloric content. Dishes span breakfast, lunch, and dinner categories. Constraints specify dietary restrictions, daily caloric limits, and variety requirements (no dish repetition across the plan).

Example dishes include *mamăligă cu brânză* (polenta with cheese), *sarmale* (cabbage rolls), *ciorbă de burtă* (tripe soup), and *salată de vinete* (eggplant salad). The combination of dietary constraints with caloric limits requires multi-step reasoning: a vegetarian meal plan with 800 calories per day significantly constrains available options.

### 3.4 Constraint checker catalog

To support replication and downstream extension, Table 2 enumerates every constraint-checker function in the released toolkit. Each entry is a pure function *f*(*W, P*, θ) → {TRUE,FALSE} over a world specification *W*, a model-produced plan *P*, and instance-specific parameters θ. Constraint instances in the released datasets are drawn from this catalog: instance generators sample a difficulty-dependent subset, choose parameter values from documented ranges, and emit the resulting list as the instance's verification target. The catalog is open-set in the toolkit: new checkers can be registered with the same signature and become available to all downstream generators without modifying scoring code. Five additional general-purpose checkers (check_days_non_empty, check_no_duplicates, check_valid_entity_ids, check_no_slot_overlaps, check_no_hallucinated_facts) are applied as structural sanity checks across all world types and are omitted from the per-world rows for brevity.

**Table 2:** Complete catalog of per-world constraint checkers in GMTW-Ro.

Function	Verifies	Parameters
Travel world (17 checkers)		
check_must_include_type	Plan contains ≥1 entity of required type	type_required
check_must_exclude_type	Plan excludes all entities of forbidden type	type_forbidden
check_exact_type_count	Plan contains exactly *k* entities of given type	type_required, exact_count
check_must_include_specific	Plan includes a specific named entity (or alias)	entity_name
check_all_family_friendly	Every selected attraction is family-friendly	—
check_budget_limit	Sum of attraction costs ≤ total budget	max_budget
check_budget_per_day	Sum of costs per day ≤ per-day budget	max_budget_per_day
check_max_total_cost	Absolute cost ceiling across the trip	max_cost
check_max_outdoor_per_day	At most *k* outdoor attractions per day	max_outdoor
check_min_activities_per_day	Each day has at least *m* entries	min_per_day
check_max_activities_per_day	Each day has at most *M* entries	max_per_day
check_max_duration_per_day	Sum of durations per day ≤ *h* hours	max_hours
check_type_diversity	Plan covers at least *T* distinct types	min_types
check_first_day_constraint	Restrictions on day 1 (indoor / outdoor / size)	indoor_only, outdoor_only, max_activities
check_last_day_constraint	Restrictions on the final day (must have outdoor; max cost)	num_days, must_have_outdoor, max_cost
check_min_total_activities	Sum of activities across all days ≥*M*	min_total
check_no_consecutive_same_type	Two consecutive days never share an identical type set	—
Schedule world (nine checkers)		
check_max_appointments_per_day	At most *M* appointments per day	max_per_day
check_max_total_appointments	At most *T* total kept appointments	max_total
check_min_total_appointments	At least *T* total kept appointments	min_total
check_keep_high_priority	All high-priority items appear in the schedule	—
check_must_drop_lowest_priority	If anything is dropped, low priorities are dropped first	—
check_priority_day_restriction	A priority level cannot be scheduled on listed days	priority, forbidden_days
check_slot_type_restriction	Appointments matching a keyword only in listed slots	type_keyword, allowed_slots
check_no_back_to_back	No single day has both morning and afternoon filled	—
check_spread_across_days	Appointments span at least *D* distinct days	min_days_with_appointments
Fact world (two checkers)		
check_answer_matches_context	Answer is consistent with the provided fact database	—
15.5-7.4,-13.5175.3mm check_exact_answer_value	Answer contains the expected value verbatim (modulo case/whitespace)	expected
Recipe world (14 checkers)		
check_all_vegetarian	Every chosen dish is vegetarian	—
check_all_vegan	Every chosen dish is vegan	—
check_no_gluten	No chosen dish contains gluten	—
check_no_lactose	No chosen dish contains lactose	—
check_max_daily_calories	Daily caloric total ≤ *C*_max_	max_calories
check_min_daily_calories	Daily caloric total ≥*C*_min_	min_calories
check_calorie_range	Daily caloric total in [*C*_min_, *C*_max_]	min_calories, max_calories
check_max_high_calorie_meals	At most *N* meals exceed a calorie threshold	calorie_threshold, max_high_calorie
check_lunch_heaviest_meal	Lunch calories strictly exceed breakfast and dinner each day	num_days
check_dinner_lightest	Dinner calories strictly below breakfast and lunch each day	num_days
check_all_meals_filled	Every breakfast/lunch/dinner slot has a dish	num_days, meals
check_max_prep_time_per_day	Sum of prep times per day ≤ limit	max_prep_time
check_quick_breakfast	Each breakfast prep time ≤ threshold	max_prep_time
check_breakfast_variety	No breakfast dish repeated across days	—

Each row lists the function name (matching the toolkit registry), the property verified against the plan, and the typed parameter(s) supplied at instance generation. Group headings indicate the world to which each block of checkers applies. Five general-purpose structural checkers (described in §3.4) are applied across all worlds and not repeated here.

### 3.5 Output format

**Figure 4:**
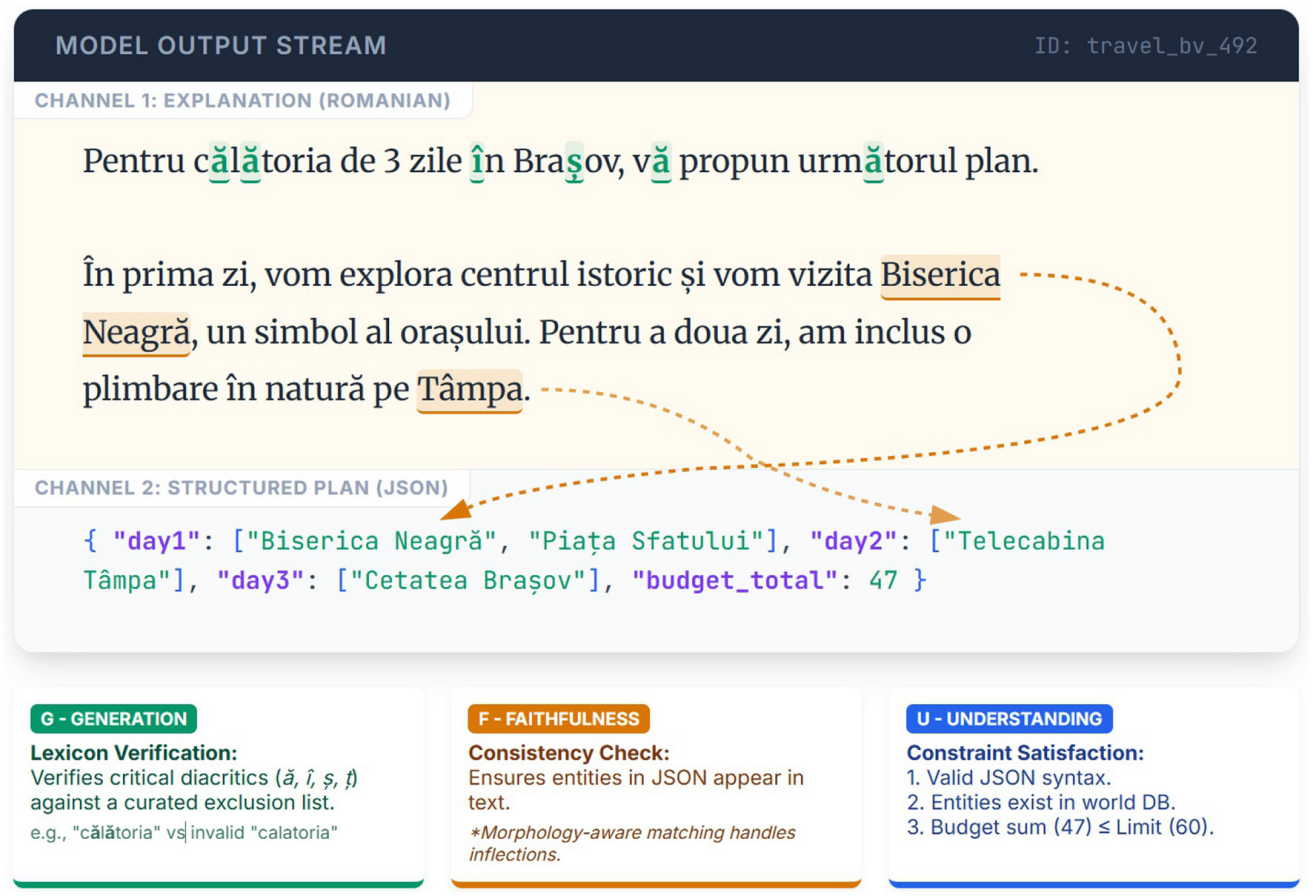
Annotated example of the dual-channel output format. The Romanian explanation (**top**) undergoes Generation quality assessment. The JSON plan (**bottom**) is checked against world constraints. Note the *Faithfulness gap*: Piaţa Sfatului and Cetatea Braşov appear in the JSON plan but are not mentioned in the explanation, reducing the *F* score for this output.

GMTW-Ro requires dual-channel outputs: a natural language explanation followed by a structured JSON plan. This ordering is strictly enforced; models producing JSON before explanation receive format penalties. The rationale is threefold. First, the explicit format requirement serves as an instruction-following test, assessing whether models can follow precise output specifications, a capability relevant to production deployments. Second, requiring explanation first ensures commitment before answer: models must articulate their reasoning before producing the structured output, preventing *post-hoc* rationalization of arbitrary selections. Third, the dual-channel structure enables evaluation separation, allowing independent assessment of linguistic quality (explanation) and planning correctness (JSON) to provide richer diagnostic signal. Figure 4 presents an annotated example of this format, illustrating how the same output is assessed along multiple dimensions.

The parser extracts both components using a robust algorithm: the JSON block is identified as the last valid JSON object in the output (accepting both markdown code fences and naked braces), with everything preceding it treated as explanation. Minor JSON syntax errors (specifically trailing commas, unclosed brackets, and unquoted keys, which LLMs commonly produce) are repaired using the deterministic json-repair library before constraint checking; outputs requiring such repair receive a 0.9 penalty on the format component (vs. 1.0 for clean parses). This library applies rule-based fixes without ambiguity, ensuring reproducibility. Complete parsing failure (malformed structure beyond simple repairs) results in null plan extraction and *U*_format_ = 0.0. While recent work has begun systematically evaluating structured output generation (Geng et al., 2025), GMTW-Ro is distinguished by its focus on semantic constraint satisfaction within the JSON content rather than schema conformance alone.

## 4 Evaluation metrics

**Figure 5:**
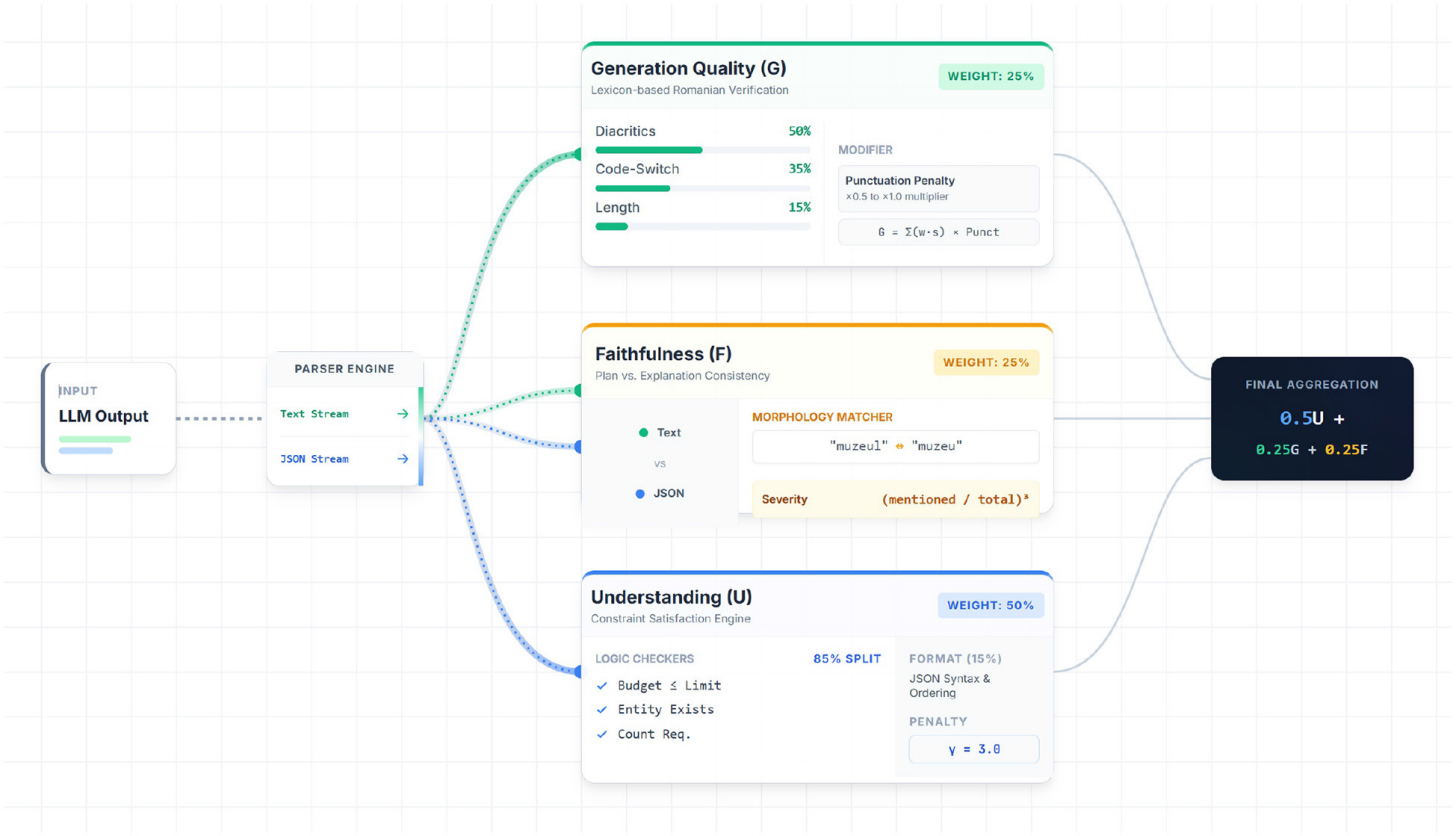
The GMTW-Ro evaluation pipeline. Model outputs are parsed into explanation and JSON components, which feed into three parallel metric computations. Understanding (U) verifies constraint satisfaction with severity penalty. Generation (G) assesses Romanian text quality through lexicon-based analysis. Faithfulness (F) checks consistency between plan and explanation using morphology-aware matching.

GMTW-Ro employs three primary metrics, each addressing a distinct evaluation dimension. Figure 5 illustrates the metric computation pipeline.

### 4.1 Understanding (U)

The Understanding metric quantifies instruction-following and constraint satisfaction. It comprises two weighted components (Equation 1):


$$\begin{array}{l} U=0.85\cdot U_{\text{constraints}}+0.15\cdot U_{\text{format}} \end{array}$$ (1)

#### 4.1.1 Constraint satisfaction (*U*_constraints_)

This component measures the proportion of instruction-level constraints satisfied by the model's plan. Each constraint corresponds to a deterministic verification function that examines the extracted JSON against the world specification. Representative checkers (see Table 2 for the complete catalog) include:

- check_budget_limit: Sum of selected entity costs ≤ total budget
- check_must_include_type: Plan contains ≥1 entity of required type
- check_max_outdoor_per_day: Count of outdoor activities per day ≤ specified maximum
- check_keep_high_priority: All high-priority appointments retained
- check_all_vegetarian / check_no_gluten: All selected dishes satisfy the stated dietary restriction.

A severity exponent amplifies the penalty for violations (Equation 2):


$$\begin{array}{l} U_{\text{constraints}}={\left(\frac{\text{satisfied}}{\text{total}}\right)}^{\gamma } \end{array}$$ (2)

where γ = 3.0 provides harsh penalization. This cubic scaling ensures that partial constraint satisfaction receives substantially reduced scores: satisfying five of six constraints yields (0.833)^3^ = 0.579, not 0.833.

The rationale for this severity penalty reflects real-world deployment requirements: constraint violations are not partial degradations of the result, but rather categorical failures. A recipe system that serves meat to a vegetarian has not achieved “83% success”; it has failed entirely, with potentially serious consequences. A travel planner that exceeds the budget renders the entire plan unusable. A scheduling assistant that double-books a time slot creates conflict rather than partial utility. Unlike accuracy metrics where 90% correct answers represent genuinely good performance, constraint satisfaction demands completeness: a model that reliably satisfies most constraints while occasionally violating critical ones is unsuitable for deployment. The cubic penalty ensures that models cannot achieve high scores through “lucky” partial compliance; consistent, complete constraint satisfaction is required.

#### 4.1.2 Format compliance (*U*_format_)

This component penalizes structural violations: missing JSON, JSON appearing before explanation, non-parseable output, or requiring extensive repair. Binary penalties apply:

- JSON missing or unparseable: *U*_format_ = 0.0
- JSON before explanation: *U*_format_ = 0.5
- Minor repairs needed: *U*_format_ = 0.9
- Clean parse with correct ordering: *U*_format_ = 1.0

The 0.5/0.9/1.0 ladder distinguishes three categories of compliance: ordering inversion (half credit, because it flips the dual-channel contract), minor repair (10% penalty, since the content is otherwise correct), and clean parse (no penalty). The 85%/15% weighting between *U*_constraints_ and *U*_format_ ensures that format compliance materially affects totals when consistently mishandled, without dominating substantive constraint satisfaction.

### 4.2 Generation quality (G)

The Generation metric assesses Romanian text quality through deterministic, lexicon-based analysis. Unlike learned quality estimators, all components use explicit word lists and rules, ensuring reproducibility and interpretability. The base score is a weighted combination of three sub-metrics (Equation 3):


$$\begin{array}{l} G_{\text{base}}=0.50\cdot G_{\text{dia}}+0.35\cdot G_{\text{cs}}+0.15\cdot G_{\text{len}} \end{array}$$ (3)

The 50/35/15 split reflects each sub-metric's diagnostic value: diacritics receive the largest weight as the most direct, unambiguous marker of insufficient Romanian competence (the ASCII-vs-diacritic distinction is binary on our curated lexicon); code-switching is weighted next because English intrusions are a documented failure mode of multilingual instruction-tuning; length contributes a small but non-zero weight to detect degenerate or empty outputs without rewarding verbosity. Punctuation quality acts as a multiplicative penalty rather than a direct component, reflecting that punctuation errors degrade overall quality proportionally (Equation 4):


$$\begin{array}{l} G=G_{\text{base}}\times P_{\text{punct}},\;P_{\text{punct}}=0.5+0.5\cdot G_{\text{punct}} \end{array}$$ (4)

Perfect punctuation (*G*_punct_ = 1.0) yields *P*_punct_ = 1.0 (no penalty); severely degraded punctuation (*G*_punct_ = 0.0) reduces the score by half. The half-floor ensures that punctuation errors alone cannot drive the Generation score to zero on an otherwise fluent Romanian explanation.

#### 4.2.1 Diacritic score (*G*_dia_)

Romanian orthography requires diacritics on specific characters. The diacritic analyzer maintains a curated lexicon of over 225 words that unambiguously require diacritical marks—cases where the non-diacritical form is never valid Romanian. This conservative set prioritizes precision (zero false positives) over recall, focusing on high-frequency function words (ş*i*, î*n, că*) and common content words that appear frequently in task-relevant text. Key examples include:

- ş*i* (and) – never valid as “si”
- î*n* (in) – never valid as “in”
- *fără* (without) – never valid as “fara”
- *călătorie* (journey) – never valid as “calatorie”

The lexicon excludes ambiguous cases where both forms are valid depending on context (e.g., *sa* could be the subjunctive *să* or the possessive *a sa*).

The score reflects the proportion of required diacritics correctly applied (Equation 5):


$$\begin{array}{l} G_{\text{dia}}=\frac{\text{correct diacritics}}{\text{correct}+\text{missing}} \end{array}$$ (5)

#### 4.2.2 Code-switch score (*G*_cs_)

Code-switching—the insertion of English words into Romanian text—indicates incomplete language separation or training data contamination. The detector employs an English word list of over 1,000 high-confidence entries unlikely to appear naturally in Romanian, including function words, discourse markers, and common vocabulary. A separate exclusion list of 200+ Romanian words protects against false positives for terms that superficially resemble English: *nu* (no), *care* (which), *vine* (comes), *mare* (sea/big).

An exponential penalty applies (Equation 6):


$$\begin{array}{l} G_{\text{cs}}=e^{-20\cdot r} \end{array}$$ (6)

where *r* is the English word rate (English words / total words). The exponential decay rate of 20 was chosen to place the half-credit threshold near *r* = 3.5% (one English token per ~30 Romanian tokens) so that a stray loanword does not dominate the score while a paragraph systematically peppered with English discourse markers does. The resulting curve tolerates minimal code-switching (one English word in 100 yields *G*_cs_≈0.82) while severely penalizing substantial contamination (10% English yields *G*_cs_≈0.14).

#### 4.2.3 Length score (*G*_len_)

Extremely short responses often indicate degenerate output, format misunderstanding, or model refusal. The length score uses a piecewise ramp requiring 100 words for full credit (Equation 7):


$$G_{\text{len}}=\{\begin{array}{ll} 0.2 & \text{if }w<10\\ 0.4+0.2\cdot \frac{w-10}{40} & \text{if }10\leq w<50\\ 0.6+0.4\cdot \frac{w-50}{50} & \text{if }50\leq w<100\\ 1.0 & \text{if }w\geq 100 \end{array}$$ (7)

where *w* is the word count. The piecewise design reflects that very short responses (<10 words) are almost certainly degenerate, moderate responses (10–50 words) provide limited but non-zero diagnostic value, and responses approaching 100 words typically contain substantive explanations. Valid explanations typically require 80–200 words to describe multi-day plans or menu selections adequately.

#### 4.2.4 Punctuation score (*G*_punct_)

Punctuation analysis detects common formatting errors:

- Space before sentence-ending punctuation (“text.” instead of “text.”)
- Missing space after punctuation (“text, next” instead of “text, next”)
- Multiple consecutive spaces
- Improper bracket/quote spacing

The score decreases with error density relative to text length, floored at 0.3 to prevent punctuation from dominating overall assessment (Equation 8):


$$\begin{array}{l} G_{\text{punct}}=\max\left(0.3,1.0-5\cdot \frac{\text{error\_count}}{\text{word\_count}}\right) \end{array}$$ (8)

Using density rather than absolute count ensures that longer outputs are not disproportionately penalized for occasional errors.

### 4.3 Faithfulness (F)

The Faithfulness metric measures consistency between the JSON plan and natural language explanation. This metric detects two failure modes: omission (planning entities without mentioning them in the explanation) and hallucination (mentioning entities not present in the plan).

The base mention score captures omission, while a multiplicative hallucination penalty discourages fabricated references (Equation 9):


$$\begin{array}{l} F={\left(\frac{\text{entities mentioned}}{\text{entities in plan}}\times 0.9^{h}\right)}^{\gamma } \end{array}$$ (9)

where *h* is the number of hallucinated entities (mentioned in the explanation but absent from the plan) and γ = 3.0 applies the same severity as the Understanding metric. The 0.9^*h*^ factor ensures that each hallucinated entity reduces the score by 10% before the severity exponent is applied. Planning three attractions but only mentioning two (with no hallucinations) yields *F* = (2/3)^3^≈0.30.

#### 4.3.1 Morphology-aware matching

Romanian's rich morphology complicates entity matching. A model might plan “Grădina Botanică” but mention “grădinii botanice” (genitive form) in the explanation. Naive string matching would miss this correspondence.

The Faithfulness analyzer employs rule-based morphology generation. For each entity, we generate approximately 15 inflected forms (the product of 2 numbers × 2–3 cases × 2 definiteness states, minus syncretized forms) covering:

- Definite article attachment: *muzeu*→*muzeul*
- Genitive/dative forms: *grădina*→*grădinii*
- Plural forms: *muzeu*→*muzee*
- Gender variations for adjectives

This covers the grammatically productive forms that appear in natural Romanian text; empirically, unmatched mentions stem from truncation or paraphrase rather than missing inflections. The explanation is searched for any generated form. This approach is deterministic and requires no external dependencies.^1^ Table 3 summarizes all metric components, their weights, and computation methods.

**Table 3:** Metric component summary.

Metric	Component	Weight	Method
U (50%)	Constraints Format	85% 15%	Checker functions Parse analysis
G (25%)	Diacritics	50%	225-word lexicon
	Code-switch	35%	1,000+ word detector
	Length	15%	Piecewise ramp (100 words)
	Punctuation	×*P*	Multiplicative penalty
F (25%)	Mention + hallucination	100%	Morphology-aware match

The three primary metrics decompose into weighted sub-components, each computed via deterministic methods requiring no learned models.

### 4.4 Final score

The composite score weights the three metrics (Equation 10):


$$\begin{array}{c} \text{Score}=0.50\cdot U+0.25\cdot G+0.25\cdot F \end{array}$$ (10)

Understanding receives the largest weight, reflecting the benchmark's emphasis on task completion over linguistic polish. A model that satisfies all constraints but produces imperfect Romanian scores higher than one producing beautiful prose that fails to follow instructions. Generation and Faithfulness contribute equally, balancing linguistic quality against internal consistency.

## 5 Solvability verification

A critical concern in constraint-based benchmarks is ensuring that generated instances admit valid solutions. An unsolvable instance would penalize models for failures outside their control, compromising evaluation validity.

GMTW-Ro addresses this through automated solvability verification. Each world type implements a backtracking solver that exhaustively searches for valid plans. Algorithm 1 gives the canonical example for the Travel world; the other three solvers follow the same structure with domain-specific constraint sets, as summarized below.

Algorithm 1 Travel-world solvability check (canonical backtracking template). Given a world specification, return True iff some assignment of *N* attractions to *N* days satisfies every constraint. Early-exit pruning is applied at the candidate-set, combination, and per-day levels so that the worst-case combinatorial enumeration is rarely realized in practice. 
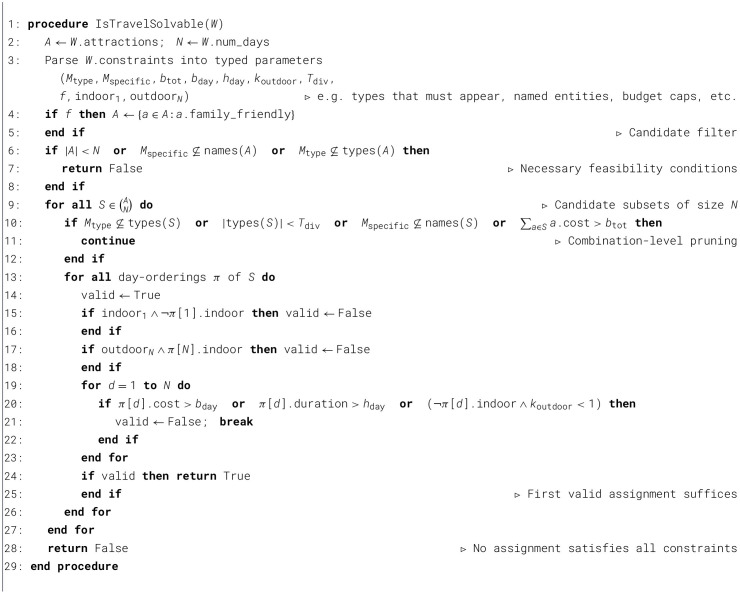

### 5.1 Travel solver

(Algorithm 1). Enumerates *N*-subsets of the (family-filtered) attraction pool, prunes any that already fail budget, type-coverage, diversity, or mandatory-inclusion checks, then verifies day-specific constraints over each permutation. Returns on the first satisfying assignment; declares unsolvable only after exhausting the search space.

### 5.2 Schedule solver

Employs constraint propagation with limited backtracking. High-priority appointments are placed first; remaining slots fill with medium/low priority respecting per-day limits. Conflict detection prevents overlapping assignments.

### 5.3 Fact solver

Verifies that the question can be answered from the provided context through direct lookup. For misbelief-trap instances, confirms that the deliberate misinformation is present and extractable.

### 5.4 Recipe solver

Applies dietary filtering to reduce candidate dishes, then checks caloric feasibility for the required meal count. Variety constraints (no repetition) are verified against filtered-set size.

During dataset generation, instances failing solvability verification are regenerated with modified seeds. The retry mechanism uses a deterministic seed transformation (original_seed + 10,000 × retry_count) to maintain reproducibility while ensuring eventual success. Empirically, over 95% of randomly generated instances are solvable; unsolvable configurations typically arise from extreme constraint combinations (very low budget with expensive mandatory types).

This verification process guarantees that every released instance admits at least one valid solution. Models receiving *U* = 0 genuinely failed to find a solution that exists.

## 6 Dataset construction

**Figure 6:**
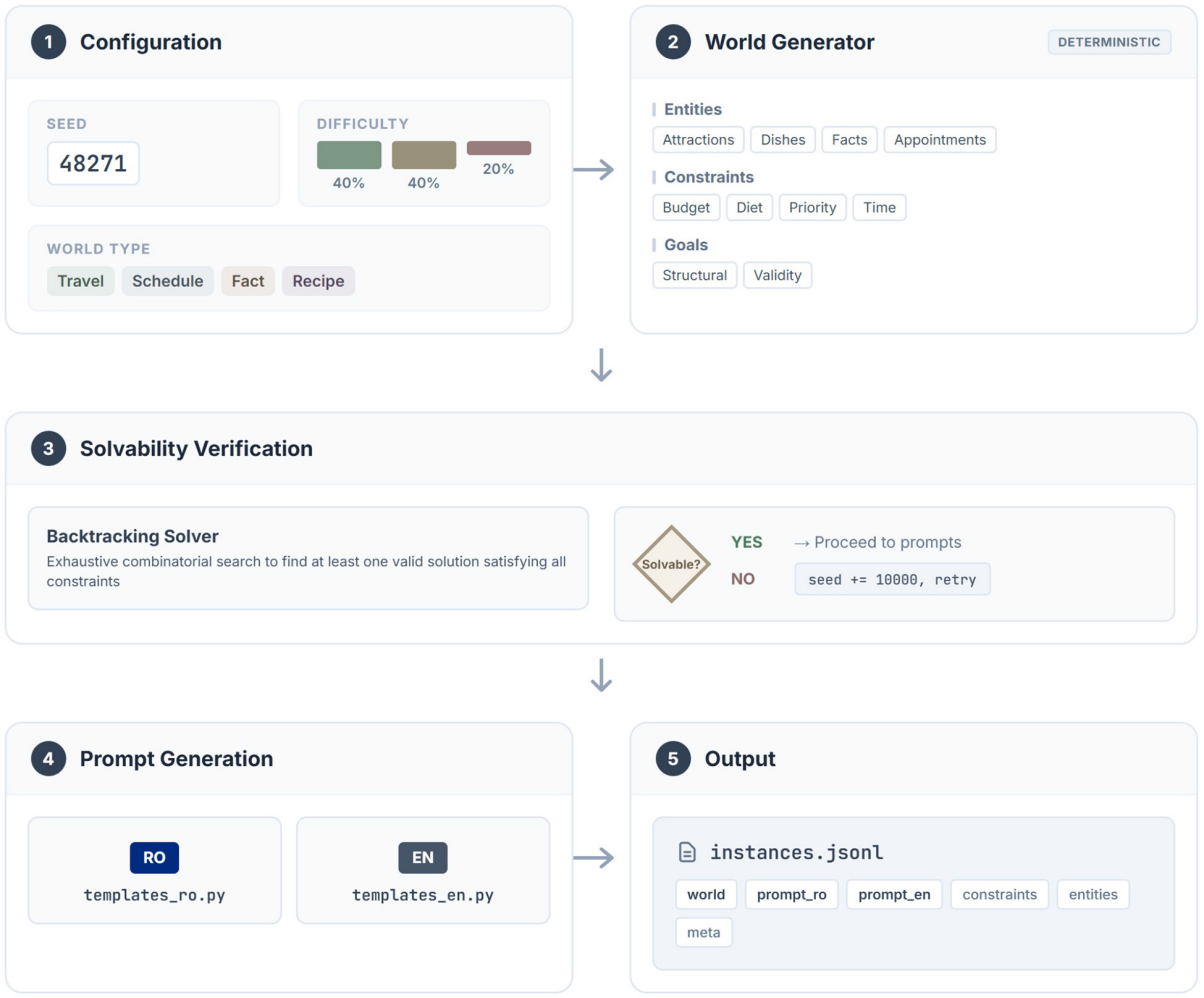
Instance generation pipeline. Configuration (seed, world type, difficulty) feeds into deterministic world generation producing entities, constraints, and goals. A backtracking solver verifies solvability; unsolvable instances trigger seed modification and retry. Verified instances receive bilingual prompt generation before output.

The complete instance generation pipeline is illustrated in Figure 6. Configuration parameters (seed, world type, difficulty) feed into deterministic world generation, with solvability verification ensuring that only valid instances reach the final dataset.

### 6.1 Standard dataset

The standard dataset comprises 500 instances distributed across world types: 200 Travel, 150 Schedule, 100 Fact, and 50 Recipe instances. The distribution is proportional to the size of each world's constraint and entity space rather than to perceived difficulty. Travel admits the richest combinations (37 attractions across six cities crossed with up to six interacting constraint types: budget bounds, type inclusion, outdoor caps, family-friendliness, multi-day allocation), and therefore receives the largest share so that sampling diversity is preserved across cities, durations, and constraint subsets. Schedule (150 instances) covers Monday–Wednesday slots with priority retention rules; Fact (100) varies misbelief-trap density across three tiers; Recipe (50), bounded by 19 dishes crossed with five dietary properties, saturates its meaningful instance space well below 100 samples, so additional instances would mostly duplicate existing configurations.

We also note explicitly that this allocation governs only the breakdown of the 500-instance standard set; the procedural generator supports unlimited additional instances per world, and the released toolkit allows researchers to construct alternative splits if their evaluation prioritizes a particular domain.

Difficulty levels follow a mixed distribution: 40% easy, 40% medium, and 20% hard. Easy instances involve 2–3 constraints with ample solution space. Medium instances increase constraint count and tighten bounds. Hard instances approach the feasibility boundary, requiring careful planning to satisfy all requirements.

Procedural generation with seeded randomness ensures reproducibility. Given identical seed values, the generation process produces identical instances, enabling version-controlled benchmarks and fair temporal comparisons.

### 6.2 Adversarial dataset

The adversarial dataset contains 300 instances designed to challenge frontier models. Key modifications include:

#### 6.2.1 Constraint density

Travel instances include 8–12 interacting constraints, compared to 3–5 in standard instances. Multiple constraints may compete for the same resources (budget, outdoor slots, required types).

#### 6.2.2 Narrow feasibility

Instances are generated near the solvability boundary, where only a few valid solutions exist. Random or greedy selection strategies are unlikely to succeed; deliberate planning is required.

#### 6.2.3 Priority conflicts

Schedule instances feature appointment sets where naive priority-following fails. Models must recognize that dropping a medium-priority appointment enables satisfying other constraints.

#### 6.2.4 Misbelief saturation

Fact instances contain 80%–100% misbelief traps, maximizing tension between context adherence and parametric knowledge. Every answer requires overriding world knowledge.

#### 6.2.5 Dietary complexity

Recipe instances combine multiple dietary restrictions (vegetarian AND gluten-free AND lactose-free) with tight caloric bounds, severely constraining valid selections.

The adversarial dataset reduces frontier model performance by approximately 10 percentage points (from ~90 to ~80%), while causing more severe degradation for weaker models.

#### 6.2.6 Disclosure on adversarial calibration

We did calibrate the adversarial generator's parameter ranges (constraint count per Travel instance, misbelief density per Fact instance, dietary-stack depth per Recipe instance) using a small set of pilot evaluations on Llama-3.3-70B and gpt-oss-20b, with the explicit goal of producing meaningful but non-saturating degradation: we wanted frontier models to drop noticeably while still retaining headroom for differentiation, rather than flooring every model at zero. The ~10pp drop on frontier models is therefore a *description* of the resulting dataset rather than an externally imposed calibration target enforced after the fact, and the per-model deltas reported in Section 9 are unconstrained by this calibration. We adopted this design choice, rather than producing maximally hard instances, because a benchmark whose adversarial split saturates uniformly at zero provides no discriminative signal for the very models we most need to differentiate.

Importantly, the standard and adversarial datasets share no instances: they are generated from disjoint seed ranges (instance IDs TRAVEL_000000–TRAVEL_000499 for standard; TRAVEL_010000+ for adversarial) and we verified $|D_{\text{std}}\cap D_{\text{adv}}|=0$ at release time. Table 4 provides detailed statistics comparing the two datasets.

**Table 4:** Dataset statistics.

Property	Standard	Adversarial
Total instances	500	300
By world type		
Travel	200 (40%)	120 (40%)
Schedule	150 (30%)	90 (30%)
Fact	100 (20%)	60 (20%)
Recipe	50 (10%)	30 (10%)
By difficulty		
Easy	200 (40%)	0 (0%)
Medium	200 (40%)	90 (30%)
Hard	100 (20%)	210 (70%)
Avg. constraints	3.8	7.2
Misbelief density	27%	85%

The standard dataset provides balanced difficulty distribution; the adversarial dataset emphasizes hard instances with increased constraint density.

## 7 Romanian NLP toolkit

The evaluation infrastructure includes a purpose-built Romanian NLP toolkit (rombench.nlp_ro). All components are rule-based and deterministic, avoiding machine learning models that could introduce variability or require external dependencies.

### 7.1 Diacritic analyzer

The diacritic lexicon was curated through linguistic analysis following three criteria:

1. Unambiguity: only words where the non-diacritical form is never valid Romanian. We exclude forms such as *sara*, which can function both as a common noun (*sară*, “evening”) and as a personal name (*Sara*).
2. Frequency: priority given to common words likely to appear in task-related text (prepositions, conjunctions, common nouns).
3. Diagnostic value: words that reliably distinguish diacritic-aware from diacritic-ignorant generation.

The analyzer handles common variations: cedilla vs. comma-below for ş and ţ (both accepted as correct), uppercase normalization, and word boundary detection. False positives from English words containing Romanian-like substrings are filtered using the exclusion list. Table 5 presents representative entries from the lexicon.

**Table 5:** Diacritic lexicon sample.

Correct Form	Invalid ASCII	English
şi	si	“and”
în	in	“in”
fără	fara	‘without”
călătorie	calatorie	“journey”
dimineaţă	dimineata	“morning”
după-amiază	dupa-amiaza	“afternoon”
grădină	gradina	“garden”
aşa	asa	“thus”

Words where the ASCII form is never valid Romanian, enabling unambiguous diacritic error detection.

### 7.2 Code-switch detector

The detector consults a curated English vocabulary of 1,111 entries (973 HIGH_CONFIDENCE_ENGLISH content words plus 138 ENGLISH_STOPWORDS) and is gated by a 202-entry Romanian LOOKALIKES exclusion list. The English vocabulary was assembled in three rounds: (i) a seed of high-frequency English function words and pronouns from the standard NLTK English stop-word inventory, (ii) discourse markers, modal verbs, and domain vocabulary observed in pilot Romanian LLM outputs (“however,” “therefore,” “budget,” “schedule,” “priority”) that were absent from the seed, and (iii) iterative expansion driven by inspecting flagged words in early model outputs to add high-frequency English content vocabulary unlikely to appear naturally in Romanian. Each candidate entry is screened against a Romanian general-purpose lexicon and rejected if the same spelling is a valid Romanian token in any reasonable register. The English word list prioritizes:

- Function words (articles, pronouns, prepositions)
- Common verbs in imperative/present forms
- Discourse markers typical of LLM outputs (“however,” “therefore,” “additionally”)
- Domain vocabulary unlikely in Romanian travel/schedule contexts

The Romanian LOOKALIKES exclusion list was built by enumerating every entry in the English vocabulary whose spelling also appears as a Romanian word, removing all such overlaps from the detection set:

- Short words overlapping with English: *a* (“has”), *o* (“a/one”), *nu* (“no”)
- Cognates with identical spelling: *general, original, special*
- Words coincidentally matching English: *vine* (“comes”), *mare* (“sea”), *are* (“has”)

#### 7.2.1 Coverage validation

To verify that the detector catches the English vocabulary that models actually produce in code-switched output, we audited it against 2,000 outputs from four models spanning the full code-switching spectrum (Qwen3-32B, which exhibits the highest English-reasoning rate; Llama-3.3-70B; RoMistral-7B; RoLlama3.1-8B). We extracted every alphabetic token of length ≥3 from each output's Romanian narrative (after stripping JSON blocks and  <think> chain-of-thought spans), compared the set against a held-out reference list of 200+ common English words known to be diagnostic of code-switching, and computed detector recall as the fraction of confirmed English tokens captured. Across these 2,000 outputs the detector identified 118,272 English tokens; only 210 tokens belonged to known English content vocabulary but were missing from the detector (three lemmas total: *take, like, look*, all observed exclusively in Qwen3's English reasoning traces and absent from the other three models), yielding an aggregate recall of 99.8%. The detector therefore captures essentially all code-switching that actually occurs in the released model outputs; the remaining gap is a fixed-cost issue (a handful of common monosyllabic English content verbs) rather than a structural blind spot.

### 7.3 Morphology generator

Romanian nouns and adjectives inflect for definiteness, case, number, and gender. The morphology generator produces variant forms through rule application:

Definite article attachment:

- Masculine: *muzeu*→*muzeul*
- Feminine: *grădină*→*grădina* (with article)
- Neuter: *parc*→*parcul*

Genitive/dative forms:

*-a* endings: *grădina*→*grădinii*
*-ă* endings: *cetate*→*cetăţii*
*-ina/-ică/-uia* special cases handled

Plural forms:

- Common patterns: *muzeu*→*muzee, parc*→*parcuri*
- Irregular forms from curated list

The generator produces approximately 15 forms per entity name, enabling robust matching without requiring ML-based lemmatization.

## 8 Cross-lingual evaluation

GMTW-Ro supports cross-lingual comparison through parallel prompts. Each instance includes both Romanian (prompt_ro) and English (prompt_en) formulations with identical constraint semantics.

English prompts use translated entity names (“Botanical Garden” for “Grădina Botanică”) and appropriate temporal vocabulary (“Monday morning” for “Luni dimineaţa”) while preserving identical constraints and world state.

The delta metric captures the “foreign language penalty,” performance degradation when models process Romanian vs. English (Equation 11):


$$\begin{array}{c} \Delta_{U}=U_{\text{en}}-U_{\text{ro}} \end{array}$$ (11)

Positive delta indicates superior English performance; values exceeding 0.10 suggest significant Romanian capability gaps warranting investigation. The Generation metric is excluded from cross-lingual comparison, as it specifically measures Romanian text quality (which English outputs would trivially fail).

This design enables researchers to distinguish:

- General reasoning failures: low U in both languages indicates constraint satisfaction difficulty independent of language.
- Romanian-specific failures: low U in Romanian but high U in English indicates language processing issues (instruction parsing, entity matching) rather than planning deficits.

### 8.1 Calibration probe

As a preliminary check that Δ_*U*_ is well-behaved on this benchmark, we measured it on the Travel world (150 instances), the domain where the constraint-checker stack is fully language-symmetric: entity attributes (cost, type, indoor/outdoor classification, family-friendliness) are stored in a language-agnostic form, so a model selecting entities by their English names in the EN prompt is scored against the same world state as a model selecting by Romanian names in the RO prompt. Three representative base open-weight models (Llama-3.1-8B-Instruct, Gemma-2-9B-it, and Gemma-7B-it) all exhibit |Δ_*U*_| ≤ 0.025, indicating that on a language-symmetric domain prompt language alone has a small and approximately neutral effect on constraint satisfaction. This is the calibration outcome we would expect of a well-translated bilingual instance set, and confirms that the released English prompts are usable for cross-lingual study without introducing systematic difficulty bias.

A full cross-lingual evaluation across all four task worlds and the 11 models reported in Section 9 requires extending the constraint catalog with language-aware variants for entity-name comparisons, in particular for the Schedule (appointment names) and Fact (database values) worlds, where the current checkers anchor on Romanian-canonical fields. We treat a dedicated study of cross-lingual deltas as a natural follow-up to this work: it would (i) extend GMTW to additional target languages so that Δ_*U*_ can be characterized across language pairs rather than only Romanian–English, (ii) develop scorer variants robust to language-asymmetric distributions of malformed structured output (a known confound for Δ_*U*_ magnitudes when a model produces format errors at different rates across its prompt languages), and (iii) systematically measure how Δ_*U*_ relates to base-model multilinguality, finetuning data composition, and inference-time language conditioning. We release the parallel English prompts alongside the benchmark to enable independent investigations along these lines.

## 9 Experimental evaluation

We evaluate 11 language models spanning different capability tiers and training approaches. The evaluation encompasses cloud-hosted frontier models accessed via API, locally-deployed open-weight models via vLLM, and Romanian-finetuned variants from the OpenLLM-Ro project. All evaluations use deterministic generation (temperature 0) with a maximum output length of 4,096 tokens to ensure reproducibility and sufficient generation capacity for detailed explanations. Table 6 presents the complete results.

**Table 6:** Model performance on GMTW-Ro standard dataset (500 instances).

Model	Deploy	*U*	*G*	*F*	JSON%	Final	95% CI
Cloud API models							
gpt-oss-20b	API	**0.929**	0.952	0.818	98%	**90.7%**	[89.3, 92.1]
Llama-3.3-70B	API	0.886	0.988	0.848	99%	90.2%	[89.0, 91.4]
Llama-4-Scout-17B	API	0.849	0.981	0.852	**100%**	88.3%	[86.9, 89.6]
Qwen3-32B	API	0.825	0.328	0.755	85%	68.3%	[66.8, 69.7]
Open-weight models (Original)							
Llama-3.1-8B-Instruct	Local	0.785	**0.995**	0.873	95%	85.9%	[84.6, 87.3]
Gemma-2-9B-it	Local	0.823	0.959	0.698	99%	82.6%	[81.0, 84.0]
Gemma-7B-it	Local	0.662	0.969	0.632	89%	73.1%	[71.4, 74.8]
Romanian-finetuned models (OpenLLM-Ro)							
RoMistral-7B-Instruct-DPO	Local	0.560	0.975	0.786	77%	72.0%	[70.1, 73.9]
RoGemma2-9B-Instruct-DPO	Local	0.546	0.837	**0.924**	70%	71.3%	[69.8, 72.9]
RoLlama3.1-8B-Instruct-DPO	Local	0.421	0.896	0.891	44%^†^	65.8%	[63.9, 67.7]
RoGemma-7B-Instruct	Local	0.318	0.858	0.851	36%^†^	58.6%	[57.3, 60.2]

Models grouped by deployment type. JSON% indicates the proportion of instances on which the model produced a valid structured output. Final = 50%U + 25%G + 25%F; 95% bootstrap CIs (2,000 resamples, fixed seed) shown in brackets. Best values per column in bold; lowest JSON% rates highlighted. ^†^Lowest JSON success rates, indicating severe structured output degradation.

### 9.1 Model selection rationale

The 11 models were chosen to satisfy three priorities: (i) every Romanian-finetuned model in the OpenLLM-Ro project must be paired with its base model, so the finetuning paradox can be measured within-family rather than across-family; (ii) the open-weight tier must span a range of base-model capability so the paradox is not architecture-specific, hence the Gemma-7B, Gemma-2-9B, and Llama-3.1-8B families and their RoGemma, RoGemma2, RoLlama adaptations; (iii) the cloud-API tier must include enough frontier models to establish a credible upper bound and verify the benchmark discriminates near the ceiling, hence Llama-3.3-70B, Llama-4-Scout-17B, gpt-oss-20b, and Qwen3-32B. We prioritized open-weight and openly served models throughout to keep evaluation reproducible by third parties. Models we considered but excluded: closed-API frontier systems such as GPT-4o and Claude (no Romanian-finetuned counterparts to pair them with, and reproducibility requires paid API access for every verifying researcher); the publicly released Mistral-7B-Instruct (the RoMistral adaptation derives from a different pretraining checkpoint, precluding a fair within-family comparison); and sub-7B models (no Ro-finetuned counterpart in any family). The toolkit is agnostic to model selection; additions by future work are straightforward and encouraged.

### 9.2 Results overview

Table 6 presents performance on the standard 500-instance dataset, while Table 7 shows results on the adversarial 300-instance dataset. Understanding scores ranged from 0.318 (RoGemma-7B) to 0.929 (gpt-oss-20b), demonstrating the benchmark's discriminative power across a 3 × performance range.

**Table 7:** Model performance on GMTW-Ro adversarial dataset (300 instances).

Model	*U*	Final	95% CI	ΔBase
Cloud API models				
Llama-3.3-70B	0.768	80.0%	78.3, 81.7	−10.2
gpt-oss-20b	0.793	79.9%	77.7, 82.0	−10.9
Llama-4-Scout-17B	0.738	79.3%	77.4, 81.0	−9.0
Qwen3-32B	0.680	60.2%	58.3, 62.0	−8.1
Open-weight models (Original)				
Llama-3.1-8B-Instruct	0.637	76.7%	74.9, 78.7	−9.2
Gemma-2-9B-it	0.721	73.3%	71.3, 75.4	−9.3
Gemma-7B-it	0.509	63.0%	60.8, 65.3	−10.1
Romanian-finetuned models				
RoMistral-7B-Instruct-DPO	0.505	68.6%	66.5, 70.7	−3.4
RoGemma2-9B-Instruct-DPO	0.531	68.0%	66.0, 70.0	−3.4
RoLlama3.1-8B-Instruct-DPO	0.374	63.3%	61.1, 65.5	−2.5
RoGemma-7B-Instruct	0.284	54.8%	53.2, 56.6	−3.8

ΔBase shows degradation from standard dataset. 95% bootstrap CIs (2,000 resamples, fixed seed) shown alongside Final. Romanian-finetuned models show smaller drops due to floor effects.

#### 9.2.1 Key finding: Romanian finetuning degrades instruction-following

The most striking result is the substantial performance degradation observed in Romanian-finetuned models compared to their base variants. We hypothesize this stems from catastrophic forgetting: the Romanian finetuning datasets likely emphasized conversational fluency without preserving instruction-following and structured output capabilities from the base models' alignment training. Llama-3.1-8B achieves 85.9% final score, while its Romanian-finetuned counterpart RoLlama3.1-8B drops to 65.8%, a 20.1 percentage point degradation. Similar patterns appear across all model families: Gemma-2-9B (82.6%) vs. RoGemma2-9B (71.3%), representing an 11.3 point drop; and Gemma-7B (73.1%) vs. RoGemma-7B (58.6%), a 14.5 point drop.

The degradation manifests primarily in two dimensions. First, Understanding scores collapse dramatically: RoLlama3.1-8B achieves only *U* = 0.421 compared to *U* = 0.785 for the original, a 46% relative decrease. Second, and more diagnostically, the ability to produce structured JSON output is severely impaired. While original Llama-3.1-8B produces valid JSON in 95% of cases, RoLlama3.1-8B succeeds only 44% of the time. This suggests the Romanian finetuning process, while potentially improving Romanian text fluency, catastrophically degraded the models' instruction-following and structured output capabilities.

**Figure 7:**
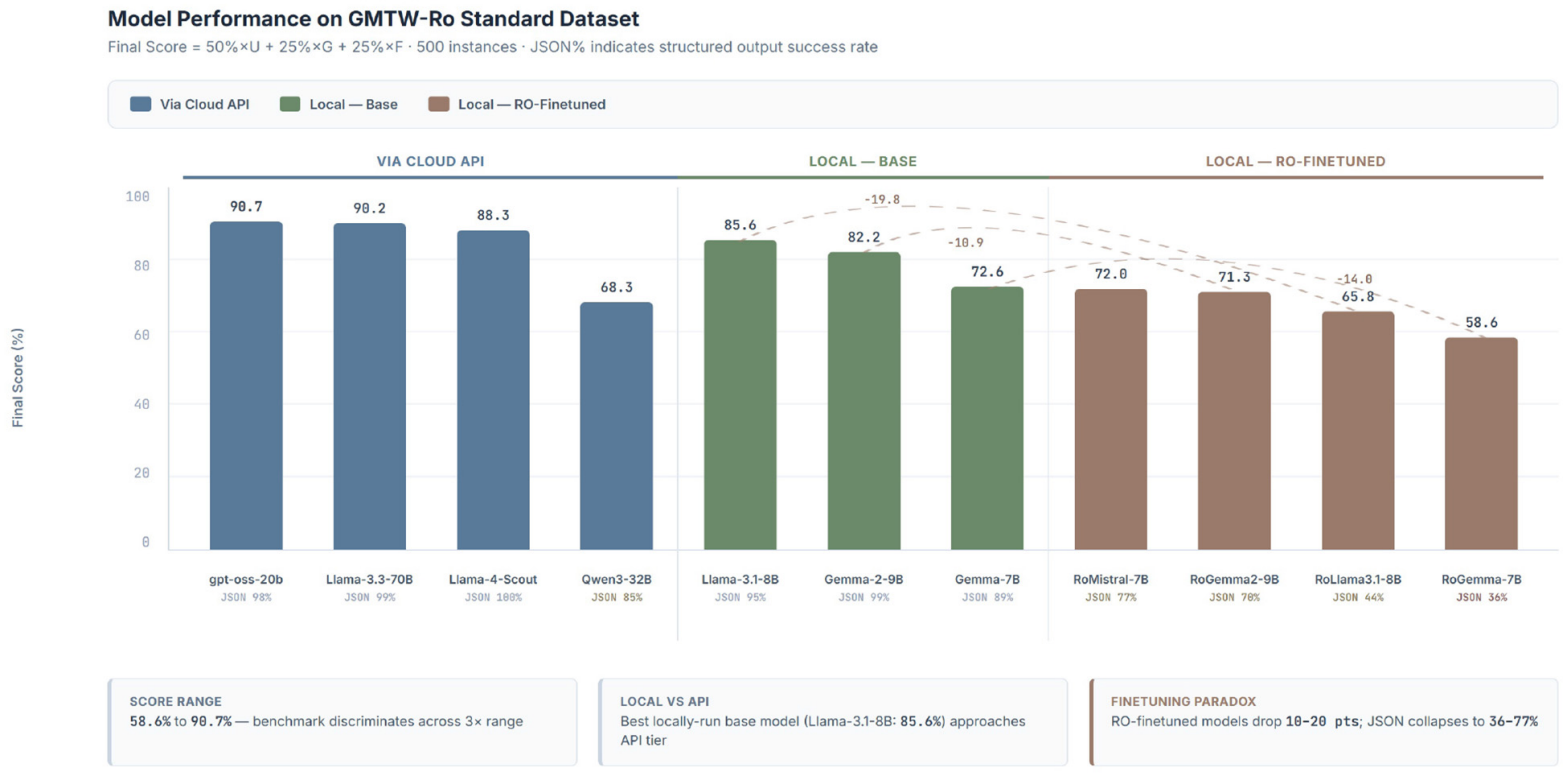
Model performance on GMTW-Ro standard dataset (500 instances). Models grouped by deployment: cloud API, locally-run base models, and Romanian-finetuned variants. Bar height indicates Final Score; JSON% below each model shows structured output success rate. Curved lines highlight the finetuning paradox: Romanian-adapted models underperform their base counterparts by 10–20 points.

Notably, Romanian-finetuned models exhibit degradation across all three metrics, including Generation quality (G). This contradicts the expectation that language-specific finetuning would improve target-language fluency: RoLlama3.1-8B achieves *G* = 0.896 vs. the base model's *G* = 0.991, and similar drops appear for both Gemma variants. The finetuning process appears to have compromised general language modeling capabilities rather than enhancing Romanian-specific performance. Figure 7 visualizes this finetuning paradox, with curved lines highlighting the consistent degradation from base models to their Romanian-adapted counterparts.

The adversarial dataset (Table 7) reveals an interesting asymmetry: frontier models experience substantial degradation (Llama-3.3-70B drops 10.2 points), while Romanian-finetuned models show minimal change (RoLlama3.1-8B drops only 2.5 points). This counterintuitive result stems from a floor effect—the Romanian models already perform poorly on the standard dataset, leaving less room for further degradation. Among API models, Qwen3-32B shows a slightly larger drop than its peers (60.2%, −8.1pp); inspection of its outputs traces this to a small fraction of adversarial instances (≈6%) on which the model's extended Romanian reasoning exhausted the available output budget before yielding valid JSON, a failure mode amplified by the heightened constraint density of the adversarial set.

### 9.3 Performance by world type

Performance varies substantially across task domains, revealing distinct capability profiles. Travel World instances, which require budget arithmetic and entity type constraints, show the clearest separation between model tiers. Frontier models achieve *U* scores above 0.85 on travel planning, while Romanian-finetuned models struggle with budget calculations, often producing plans that exceed stated limits.

Schedule World presents unique challenges due to Romanian temporal vocabulary. Models must correctly parse and reproduce day names (*Luni, Marţi, Miercuri*) and time slots (*dimineaţă, după-amiază*) with proper diacritics. Errors in temporal parsing cascade into structural failures, as incorrectly named calendar slots cannot be validated. This domain particularly exposes diacritic handling: models that strip or corrupt diacritics fail format checks even when their logical planning is sound.

Fact World instances, which test context adherence through deliberate “misbelief traps,” reveal the tension between parametric knowledge and instruction-following. When asked questions whose answers contradict common knowledge (e.g., context stating “Capitala României: Sibiu” when the actual capital is Bucharest), models must subordinate world knowledge to provided context. Preliminary analysis suggests that smaller models more readily override context with parametric knowledge, while larger models demonstrate better context adherence, though none achieve perfect trap avoidance.

Recipe World, though comprising the smallest instance count, imposes dense constraint interactions. Dietary restrictions (vegetarian, vegan, and gluten-free) combined with caloric limits require simultaneous multi-constraint reasoning. Models must correctly identify which dishes satisfy which restrictions from the Romanian dish descriptions, then ensure daily caloric totals remain within bounds. This domain shows the highest variance in *U* scores across models.

### 9.4 Diacritic and code-switching patterns

The Generation metric reveals systematic patterns in Romanian text quality. We analyze two primary components: diacritic correctness (*G*_dia_) and code-switching absence (*G*_cs_).

#### 9.4.1 Diacritic analysis

Original English-trained models achieve near-perfect diacritic scores (*G*_dia_>0.99), demonstrating that their training data included properly diacriticized Romanian. Surprisingly, Romanian-finetuned models show lower diacritic scores in several cases: RoGemma2-9B achieves *G*_dia_ = 0.941 compared to Gemma-2-9B's 0.998. This suggests the Romanian finetuning corpus may have contained inconsistently diacriticized text.

The most commonly missed diacritics involve the letters ş (s-comma) and ţ (t-comma). Some models produce cedilla variants (ş with cedilla) instead of comma-below variants (ş with comma), though our analyzer accepts both as correct per Unicode normalization. The diagnostic pair ş*i*/“si” proves most revealing: correct usage indicates proper Romanian tokenization, while “si” suggests the model treats Romanian as ASCII-approximated text.

#### 9.4.2 Code-switching detection

Frontier models exhibit minimal code-switching (*G*_cs_>0.98), maintaining language separation throughout generation. The notable exception is Qwen3-32B, which achieves only *G*_cs_ = 0.100 on the standard dataset due to extensive English reasoning traces before Romanian output. Common English intrusions across models include discourse markers (“however,” “therefore,” “additionally”) and planning vocabulary (“budget,” “schedule,” “priority”). These patterns suggest that instruction-tuning in English creates persistent associations between reasoning tasks and English vocabulary, even when explicitly requested to respond in Romanian.

### 9.5 Faithfulness analysis

Counter-intuitively, Romanian-finetuned models achieve higher Faithfulness scores than their base counterparts. RoLlama3.1-8B attains F=0.891 compared to Llama-3.1-8B's *F* = 0.885; RoGemma2-9B achieves *F* = 0.924 vs. Gemma-2-9B's markedly lower *F* = 0.698. This pattern admits a sobering interpretation: Romanian-finetuned models often generate simpler plans with fewer entities, making full mention in the explanation easier. When a model plans only two attractions (often due to instruction-following failures), mentioning both is trivial compared to correctly referencing six attractions in a well-constructed itinerary.

We identify three common Faithfulness failure modes. First, *generic explanations* rely on template-like justifications [e.g., “*Am ales aceste locuri pentru că sunt interesante*” (“I chose these places because they are interesting”)] that lack entity-specific grounding. Second, *partial mention* occurs when complex plans with 5+ entities see decreased mention rates; models often describe the first few planned items in detail, then truncate or summarize the remainder. Third, *morphological mismatch* persists despite our inflection-aware matching when entities are referenced using unexpected or underspecified forms, for instance, “Muzeul de Artă” referenced simply as “muzeu” without the definite article, which our strict matching correctly flags as unmentioned.

### 9.6 Sensitivity analysis

To verify that our model rankings and headline conclusions are not artifacts of specific parameter choices, we ran a sensitivity analysis over the four hardcoded scoring constants: the severity exponent γ, the code-switch decay rate *k* in $G_{\text{cs}}=e^{-k\cdot r}$, the Final-score weights (*w*_*U*_, *w*_*G*_, *w*_*F*_), and the per-hallucination factor α in the Faithfulness formula. For each perturbation we re-aggregated the per-instance scores (no new model calls were required; the released metrics contain the raw component values) and compared the resulting Final scores and rankings against the canonical configuration. Table 8 reports the mean and maximum cell shifts together with Spearman rank correlations against the canonical ranking.

**Table 8:** Sensitivity of model rankings to scoring constants.

Variant	Mean Δ (pp)	Max |Δ|(pp)	Spearman ρ
**Canonical** (γ = 3, *k* = 20, weights 50/25/25, α = 0.9)	0.00	0.00	1.000
Severity exponent			
γ = 1 (linear, no severity)	+8.07	11.92	0.945
γ = 2 (quadratic)	+3.22	4.52	0.991
γ = 4 (stricter)	−2.24	2.99	1.000
Code-switch decay rate			
*k* = 10 (softer)	+0.27	1.88	1.000
*k* = 40 (harsher)	−0.22	0.78	1.000
Final-score weights (*w*_*U*_/*w*_*G*_/*w*_*F*_)			
60 / 20 / 20 (U-heavy)	−0.87	3.68	0.991
40 / 30 / 30 (less U-heavy)	+0.87	3.68	0.982
16-7.4,-13.5242pt70 / 15 / 15 (strong U)	−1.74	7.36	0.964
Hallucination factor			
α = 1.0 (no penalty)	+2.31	3.67	1.000
α = 0.7 (stricter)	−2.09	2.88	0.991

Each row perturbs one parameter relative to the canonical configuration (in bold); other parameters are held fixed. “mean Δ” is the average Final-score change across the 11 models in percentage points; “max |Δ|” is the largest such shift on any single model; Spearman ρ is computed against the canonical ranking. Rankings are preserved at ρ≥0.945 for every variant, including the most aggressive ablation that removes the severity exponent entirely.

Three observations are worth highlighting. First, the severity exponent γ is the single most influential constant: relaxing it to γ = 1 shifts mean Final scores by +8.07 pp and the worst-affected cell by 11.92 pp. This is the expected mechanical effect of removing the cubic penalty for partial constraint satisfaction; nonetheless, even this most aggressive perturbation preserves the ranking at Spearman ρ = 0.945, with the finetuning-paradox direction (Ro-finetuned models below their base counterparts) intact in every pairwise comparison. Second, the code-switch decay rate *k* is essentially inert for rankings (Spearman ρ = 1.000 at *k*∈{10, 40}), because *G* is a 25% weight in the Final score and code-switching itself is rare for most models. Third, the U/G/F weight scheme is fairly stable: even a 70/15/15 “strong-U” weighting only moves cells by up to 7.36 pp with ρ = 0.964. Across all 11 tested perturbations, the qualitative conclusions of the paper (rankings, the ~10pp finetuning-paradox magnitudes, and the floor effect on the adversarial split) are robust to reasonable choices of the scoring constants. The scoring decisions made by the benchmark designer therefore influence the precise numerical values, as one would expect of any aggregation, but do not drive the model ordering.

### 9.7 Error analysis by failure type

Aggregate Final scores compress several qualitatively distinct failure modes into a single number, obscuring the diagnostic signal the benchmark is designed to surface. To make this signal explicit, Table 9 decomposes each model's failures into five distinct signals computed deterministically from the released metrics: *malformed JSON* (the output cannot be parsed even after repair), *constraint violations* (the parse succeeds but at least one constraint is unsatisfied), *code-switching* (the Romanian narrative contains more than 1% English tokens), and two Faithfulness modes, *omission* (an entity in the plan is not mentioned in the explanation) and *hallucination* (an entity in the explanation does not appear in the plan).

**Table 9:** Failure-type decomposition on the standard 500-instance set.

Model	malf.%	constr.%	cs%	omit%	hall.%
Cloud API					
gpt-oss-20b	1.0	10.2	5.8	0.0	23.4
Llama-3.3-70B	0.6	23.2	2.4	0.0	21.0
Llama-4-Scout-17B	0.0	31.4	1.6	0.0	28.6
Qwen3-32B	17.0	11.8	100.0	0.0	38.6
Open-weight base					
Llama-3.1-8B	0.0	42.8	0.4	4.2	30.6
Gemma-2-9B	0.0	35.8	2.2	27.6	27.0
Gemma-7B	1.2	55.4	3.0	30.0	25.0
Romanian-finetuned					
RoMistral-7B	1.4	64.0	3.2	13.0	28.8
RoGemma2-9B	31.6	36.0	22.8	0.2	19.8
RoLlama3.1-8B	68.8	14.2	1.8	0.0	8.4
RoGemma-7B	35.2	41.4	8.0	0.0	22.8

Columns report the percentage of instances exhibiting each failure mode independently (a single instance can contribute to several columns; e.g., a malformed-JSON output also fails constraint checking by definition). malf.% = parse failed beyond repair; constr.% = parse succeeded but ≥1 constraint violated; cs% = English token rate >1%; omit% = plan entities not mentioned in explanation; hall.% = explanation entities absent from plan.

The decomposition reveals that the three model tiers fail in qualitatively different ways. Cloud API models almost never produce malformed JSON ( ≤ 1% for three of four) and have low constraint-violation rates; their dominant residual failure mode is hallucination (23%–29%), reflecting the tendency to generate plausible-sounding entities consistent with the world's domain but not present in its specific catalog. Qwen3-32B is an outlier among API models because of its English-language reasoning traces, visible in the 100% code-switching rate. Open-weight base models retain clean JSON output but show markedly higher constraint-violation rates (36%–55%); they also exhibit substantial omission rates (27%–30% for the Gemma family), indicating that their explanations rarely reference the specific entities chosen in the plan. Romanian-finetuned models show the most distinctive pattern: structured output capability collapses (malformed JSON jumps to 32%–69% for three of four Ro-models) while constraint-violation rates among the parseable subset remain comparable to base counterparts, suggesting the language adaptation primarily damages output-format adherence rather than the model's ability to reason about constraints given a successful format.

#### 9.7.1 Qualitative classification

The five failure-mode columns map naturally onto three higher-level error categories that are useful for diagnosing where remediation should focus: (a) *bad formatting*, captured by the malformed-JSON column, where the model fails to produce parseable structured output, regardless of whether its underlying reasoning was correct; (b) *grounding/alignment failures*, captured by the constraint-violation and hallucination columns, where the model produces parseable output that contradicts the world specification (an entity outside the catalog, a budget exceeded, a forbidden type included); and (c) *insufficient justification*, captured by the omission column, where the structured plan may be correct but the natural-language explanation fails to ground itself in the chosen entities, signaling that the model produced a plan without articulating why. The three Romanian-finetuned models with high malformed-JSON rates (RoLlama, RoGemma2, RoGemma) fail primarily through (a); the Gemma family base models fail primarily through (c) for the omission signal but also through (b) on constraints; the frontier API models fail primarily through (b) via hallucination. These signatures suggest different interventions are warranted for each cluster: structured-output fine-tuning for the Ro-models, entity-grounding training for the open-weight base models, and tighter context fidelity for the frontier API models.

## 10 Extending GMTW

While our implementation targets Romanian, the GMTW architecture generalizes to other languages. We provide documentation and extension points for adaptation:

### 10.1 Adding new languages

Adapting GMTW to a new language requires:

1. Prompt translation: convert system prompts and constraint descriptions. The modular prompt structure isolates translatable content.
2. Entity localization: create culturally appropriate entities (cities, dishes, appointment types) with authentic names.
3. Diacritic lexicon: for languages with diacritics (Turkish, Vietnamese, Polish, etc.), curate a list of unambiguous diacritic-requiring words.
4. Code-switch detector: adapt the English word list and create a language-specific exclusion list for cognates and false friends.
5. Morphology rules: implement inflection generation for the target language's grammatical features.

The core evaluation logic (constraint checking, JSON parsing, and metric computation) requires no modification.

### 10.2 Adding new world types

Researchers can extend GMTW with additional task domains by implementing:

1. World generator: a class that produces instances with configurable difficulty, including prompt templates and constraint specifications.
2. Constraint checkers: functions that verify specific requirements against extracted plans.
3. Solvability prover: a solver (backtracking, constraint propagation, or domain-specific algorithm) that verifies instance feasibility.
4. Entity database: a curated set of domain-appropriate entities with relevant properties.

Potential extensions include: transportation planning (routes, schedules, and connections), event planning (venues, catering, and timelines), or educational curriculum design (courses, prerequisites, and credit limits).

## 11 Discussion

### 11.1 Benchmark scope and limitations

GMTW-Ro targets a specific evaluation objective: the deterministic assessment of constraint-following ability in Romanian. This design choice entails several deliberate limitations.

The benchmark does not assess open-ended generation, creative writing, or conversational competence. All tasks are structured and defined by explicit success criteria. Consequently, models that perform well on GMTW-Ro may nonetheless struggle with less constrained Romanian-language tasks. Conversely, models capable of producing fluent and stylistically rich Romanian prose may fail under GMTW-Ro's strict constraint requirements.

The diacritic lexicon, while carefully curated, covers only a subset of Romanian vocabulary. Tokens absent from the lexicon are excluded from diacritic evaluation, potentially underestimating errors involving rare words, neologisms, or domain-specific terminology. Similarly, the code-switch detector may fail to identify uncommon English words, proper nouns, or novel lexical borrowings.

Faithfulness is measured through morphology-aware string matching, which improves upon naive substring-based approaches but remains inherently limited. A model may accurately convey the intended information using synonyms or paraphrases yet still incur penalties for failing to explicitly mention planned entities. This limitation reflects the benchmark's emphasis on determinism: incorporating semantic similarity judgments would require learned representations and introduce additional sources of variability.

#### 11.1.1 Contamination and overfitting

Benchmark contamination (memorisation of evaluation instances during training) is mitigated structurally rather than through obfuscation. The released 500+300 instances are one realization of a procedural generator parameterised by a random seed; researchers can produce arbitrarily many additional, non-overlapping instances by varying the seed, with the toolkit exposing the generator as a first-class API. Any model that overfits to the released split can therefore be re-evaluated on a freshly sampled split of the same size and difficulty, with the same world types, entity inventories, and constraint catalog (Table 2) but disjoint world-IDs and constraint configurations. Because diversity is bounded by this fixed combinatorial structure rather than by stochastic prompt mutation, re-sampled splits inherit the same distribution of failure modes that the released datasets exercise, preserving comparability while making static memorisation an unstable strategy.

### 11.2 The knowledge-behavior gap revisited

Our experimental results confirm the knowledge-behavior gap hypothesis. Models that achieve strong Generation scores—producing fluent Romanian with proper diacritics—do not necessarily achieve high Understanding scores, which reflect constraint satisfaction. This decoupling indicates that surface-level language competence (vocabulary, grammar, orthography) is insufficient for successful task execution.

Results from the Fact World tasks are particularly revealing. Models readily answer questions when the provided context aligns with their parametric world knowledge, yet consistently struggle with misbelief traps. This behavior suggests a failure to subordinate internal knowledge to externally supplied context, a capability crucial for retrieval-augmented and tool-using systems but difficult to probe using traditional language benchmarks.

### 11.3 Implications for Romanian NLP

Results on GMTW-Ro highlight several important directions for improving Romanian language model capabilities.

#### 11.3.1 The Romanian finetuning paradox

The most striking finding is that Romanian-finetuned models underperform their base counterparts on GMTW-Ro by 10–20 percentage points. This degradation warrants closer examination of the effects of language adaptation on structured reasoning and instruction-following.

The degradation is not uniform across task domains, and the per-domain U decomposition is informative about which capabilities are being lost. For the Llama-3.1-8B family, the largest drops occur on Travel (−54.7pp: 0.766 → 0.219) and Recipe (−47.0pp: 0.777 → 0.307), both domains demanding multi-day constraint juggling over a fixed entity inventory, while Fact suffers the smallest drop (−23.0pp), indicating that single-fact context retrieval is the most robust capability across the adaptation. The Gemma-2-9B family shows a different signature: Fact is hit hardest (−40.0pp: 0.992 → 0.592), suggesting that the RoGemma2 adaptation specifically degrades the model's willingness to subordinate parametric knowledge to provided context: models that should answer “Frankfurt” (the misbelief-trap answer) instead fall back on “Berlin” (their world-knowledge answer). For the Gemma-7B family, both Fact (−47.6pp) and Travel (−37.7pp) collapse, while Schedule drops only −22.7pp; Schedule is structurally the simplest domain (fewer entity types, more uniform constraints), and it is the only domain that degrades comparably across all three model families. This heterogeneity argues against any single-cause explanation: different finetuning pipelines damage different downstream capabilities, and the constraint-rich Travel and context-adherence Fact domains are the primary stress tests where each family's weakness becomes visible.

Aggregated across these domains, three dominant failure patterns are visible in the outputs: (1) *format collapse*, in which models generate conversational Romanian responses without the required JSON structure, often acknowledging the task but failing to produce structured output; (2) *instruction drift*, where models satisfy a subset of constraints while ignoring others, particularly struggling with negation constraints (e.g., “nu include…”/“do not include…”) and compound requirements; and (3) *entity hallucination*, whereby models reference plausible-sounding entities absent from the provided world state, indicating insufficient grounding to context.

Concrete examples illustrate these failure modes. In one Travel World instance, a model states in its explanation “*am încercat să includ un monument istoric*” (I tried to include a historic monument), yet the generated plan contains no such entity, violating the explicit constraint. In another case, the model hallucinates “Muzeul de Etnografie” and “Telecabina de la Muntele Tâmplăriei,” plausible-sounding Romanian attractions not present in the world state. A budget-arithmetic failure pattern is also frequent: RoLlama plans regularly include four attractions totalling 180 RON against a stated budget of 100 RON, even when the explanation explicitly states “*respectând bugetul stabilit*” (respecting the established budget), indicating that the model has the linguistic competence to acknowledge the constraint but has lost the procedural competence to satisfy it. A more subtle failure concerns Romanian typographic conventions: some outputs use Romanian quotation marks instead of ASCII quotes within JSON structures, resulting in syntactically invalid output despite otherwise correct structural intent. Even when normalizing such quotes prior to evaluation (a lenient adjustment, given that valid JSON requires ASCII quotes), the resulting improvement is modest (2.7 percentage points for RoGemma-7B), indicating that the primary source of degradation lies in constraint satisfaction rather than surface-level formatting.

Several hypotheses may explain this phenomenon. First, instruction tuning in a non-native language may impose a “capability tax,” adapting models to follow instructions in Romanian could inadvertently degrade general reasoning or structured output abilities acquired during base training. Second, optimization toward specific language benchmarks (common in language adaptation efforts) may improve targeted metrics while harming out-of-distribution performance on tasks that require precise adherence to constraints. Third, the data used for language adaptation naturally prioritizes conversational fluency over structured task completion, whereas GMTW-Ro explicitly evaluates the latter.

Taken together, these findings suggest that future Romanian language adaptation efforts should explicitly monitor and preserve instruction-following and structured output capabilities throughout the finetuning process, rather than optimizing solely for surface-level linguistic proficiency.

#### 11.3.2 Structured output preservation

The collapse in JSON output success rates (from 95 to 44% for Llama variants) constitutes a specific and measurable failure mode. Romanian finetuning pipelines should therefore include explicit evaluation of structured output capability both before and after finetuning, with automatic rejection of checkpoints that degrade this capability beyond predefined thresholds.

#### 11.3.3 Training data quality

Diacritic inconsistencies across models likely stem from deficiencies in training data. Romanian web text frequently omits diacritics; models trained on such data inherit these errors. Notably, several Romanian-finetuned models exhibit worse diacritic handling than their base counterparts, suggesting that the finetuning corpus itself contained improperly diacriticized text. Careful data curation emphasizing correct Romanian orthography is therefore essential.

#### 11.3.4 Code-switching patterns

The persistent insertion of English discourse markers (e.g., “however,” “therefore”) into otherwise Romanian text indicates incomplete language separation during training. Targeted training on strictly monolingual Romanian corpora, combined with explicit penalties for code-switching during RLHF, may help mitigate this behavior.

#### 11.3.5 Instruction-following in Romanian

Constraint satisfaction degrades substantially as the number and complexity of constraints increase. The performance gap between base and Romanian-finetuned models widens on complex, multi-constraint tasks, suggesting that existing Romanian instruction-tuning datasets may lack sufficient coverage of complex, multi-step instructions. Translated English instruction datasets [as advocated in prior work (Masala et al., 2024b)] may help address this gap, provided that instruction-following capabilities are explicitly preserved during language adaptation.

## 12 Conclusion

GMTW-Ro provides a rigorous framework for evaluating Romanian language model capabilities through grounded task worlds, deterministic verification, and decomposed metrics. By design, the benchmark enables precise diagnosis of model strengths and weaknesses without reliance on human annotators or auxiliary models.

Our evaluation of 11 models yielded a critical finding: Romanian-finetuned models perform substantially worse than their base variants on GMTW-Ro, with degradations of 10–20 percentage points. For example, RoLlama3.1-8B model achieves only 65.8% overall accuracy compared to Llama-3.1-8B's 85.9%, while structured JSON output success rates drop from 95 to 44%. This *Romanian finetuning paradox* raises fundamental questions about whether language adaptation intrinsically trades general reasoning and instruction-following capabilities for target-language fluency, or whether current methodologies simply fail to preserve these capabilities—an issue with immediate implications for multilingual NLP research.

By emphasizing constraint satisfaction rather than surface fluency, GMTW-Ro exposes failures that fluency-oriented evaluations overlook. Models may generate grammatical Romanian while failing to follow complex instructions; GMTW-Ro makes this gap explicit. Across all task domains, original English-trained models (Llama-3.1-8B, Gemma-2-9B) consistently outperform their Romanian-finetuned counterparts suggesting that frontier multilingual models may already possess sufficient Romanian competence for structured task-solving, while poorly designed language adaptation can induce regression.

Our research contributions are twofold: (1) the release of curated evaluation datasets comprising 500 standard and 300 adversarial instances, all verified solvable, alongside empirical evidence demonstrating that Romanian finetuning requires careful design to avoid capability degradation; and (2) a decomposed deterministic metric framework (Understanding, Generation, and Faithfulness) enabling fine-grained capability analysis without learned judges. These are accompanied by a purpose-built Romanian NLP toolkit (diacritic lexicon with over 225 unambiguous entries, code-switch detector covering more than 1,000 words) and a language-agnostic infrastructure released to support extension to additional languages.

Future work will expand the diversity of world types, investigate parameter-efficient finetuning methods that better preserve instruction-following, and develop Romanian instruction datasets explicitly targeting structured output tasks. We invite the research community to contribute instances, constraint types, and evaluation components. The complete toolkit is available at https://github.com/AndreiBulzan/gmtw-hria.

## Data Availability

The datasets presented in this study can be found in online repositories. The names of the repository/repositories and accession number(s) can be found below: https://github.com/AndreiBulzan/gmtw-hria.

## Footnotes

1 Optional extensions for Stanza-based lemmatization and LanguageTool grammar checking are available in the toolkit but not used in reported evaluations to maintain full determinism.
