# Supplementary material for: GMTW-Ro: a deterministic benchmark for evaluating large language models on grounded Romanian tasks
Source: Front Artif Intell. 2026 Jul 2;9:1831918. doi: 10.3389/frai.2026.1831918 (PMC13372750; doi:10.3389/frai.2026.1831918)
Supplement: Supplementary file 1 [file Data_Sheet_1.PDF]

Supplementary Material

1 S1: DIACRITIC LEXICON SAMPLE BY CATEGORY

The G_{dia} metric uses a curated lexicon of Romanian words where the ASCII (non-diacritical) form is *never* valid. Table S1 shows representative entries by category. The complete lexicon (225+ unambiguous entries) is available at `rombench/nlp_ro/lexicon.py`.

Table S1. Diacritic Lexicon Sample by Category. Representative entries from the 225+ word lexicon used for G_{dia} computation. Full lexicon available in the toolkit.

Category	Examples (ASCII → correct form)
Function words	si→şi, in→în, daca→dacă, fara→fără
Conjunctions	insa→însă, inca→încă, asadar→aşadar
Quantity	cat→cât, cati→câţi, cateva→câteva
Temporal	maine→mâine, intai→întâi, marti→marţi
Verbs (în- prefix)	incepe→începe, intelege→înţelege
Verbs (şti- stem)	stie→ştie, stiu→ştiu, stiinta→ştiinţă
2nd person plural	faceti→faceţi, aveti→aveţi, sunteti→sunteţi
Nouns (-ţie suffix)	functie→funcţie, atentie→atenţie
Common nouns	tara→ţară, oras→oraş, paine→pâine

2 S2: PROMPT TEMPLATES

This section presents example prompts for all four task worlds. The Travel World prompt (Figure S1) shows budget and activity-type constraints. The Fact World prompt (Figure S2) demonstrates a "misbelief trap"—context that deliberately contradicts world knowledge. The Schedule World prompt (Figure S3) illustrates calendar organization with priority constraints. The Recipe World prompt (Figure S4) shows dietary restriction and caloric limit handling.

Examining these prompts reveals how each task world creates distinct cognitive demands while sharing a common evaluation structure. Travel World requires arithmetic reasoning, as the model must sum admission costs across multiple days while simultaneously tracking activity-type quotas and indoor/outdoor balances. Schedule World tests temporal reasoning and priority hierarchies, requiring the model to recognize that retaining all high-priority appointments may necessitate difficult trade-offs against per-day limits. Fact World probes the boundary between parametric knowledge and contextual instruction-following by deliberately presenting familiar-sounding but incorrect facts; a model that cannot subordinate its learned knowledge to explicit context will fail these instances. Recipe World combines categorical filtering (identifying dishes that satisfy dietary restrictions) with numerical constraints (caloric totals), requiring reasoning about properties that may not be explicitly stated. For example, the model must recognize that *Sarmale* contains meat based on the absence of a vegetarian tag, even when the prompt displays only caloric information.

Across all worlds, the strict explanation-then-JSON ordering serves a diagnostic purpose: models must commit to a reasoning narrative before producing the structured answer, preventing post-hoc rationalization

Romanian (original)

```
Ai 3 zile la dispoziţie în Braşov
pentru o excursie.
Ai următoarele opţiuni de
vizitare:
 • Biserica Neagră (monument,
interior, potrivit pentru copii,
25 lei)
 • Parcul Central (parc, exterior,
potrivit pentru copii, 0 lei)
 • Cetatea Braşov (monument,
exterior, potrivit pentru copii,
30 lei)
 • Muzeul de Artă (muzeu, interior,
nu este potrivit pentru copii
mici, 20 lei)
Te rog să:
1. Creezi un plan pentru cele 3
zile, în format JSON.
2. Scrii o explicaţie în limba
română (2-3 paragrafe).
Trebuie să respecţi următoarele
cerinţe:
 - Bugetul total să nu depăşească
80 lei
 - Include cel puţin un monument
 - Maximum 2 activităţi în
exterior
IMPORTANT: ÎNTÂI scrie explicaţia,
APOI blocul JSON.
```

English (translation)

```
You have 3 days available in
Braşov for a trip.
You have the following visiting
options:
 • Black Church (monument, indoor,
child-friendly, 25 lei)
 • Central Park (park, outdoor,
child-friendly, 0 lei)
 • Braşov Fortress (monument,
outdoor, child-friendly, 30 lei)
 • Art Museum (museum, indoor, not
suitable for small children, 20
lei)
Please:
1. Create a plan for the 3 days,
in JSON format.
2. Write an explanation in
Romanian (2-3 paragraphs).
You must respect the following
requirements:
 - Total budget must not exceed 80
lei
 - Include at least one monument
 - Maximum 2 outdoor activities
IMPORTANT: FIRST write the
explanation, THEN the JSON block.
```

Figure S1. Example Travel World prompt (bilingual). The model receives attractions with properties, explicit constraints, and format requirements. All constraints are programmatically verifiable.

of arbitrary selections. The consistent embedding of Romanian diacritics in both natural language and JSON keys (e.g., `Luni_dimineaţă`, `day1_prânz`) ensures that structured output evaluation captures orthographic competence alongside logical correctness.

3 S3: EXTENDED RESULTS

Table S2 presents a summary comparison between original and Romanian-finetuned models, highlighting the degradation pattern across all model families tested.

The consistent pattern of degradation across all three model families—with Understanding scores dropping 34–52% and JSON success rates dropping 29–53%—suggests a systematic issue with current Romanian finetuning methodologies rather than model-specific factors.

Romanian (original)

```
Ai la dispoziţie următoarea bază
de date cu informaţii:
 • Capitala României: Sibiu
 • Cel mai lung râu: Dunărea
 • Cel mai înalt vârf: Moldoveanu
 • Populaţia: 19 milioane
ATENŢIE: Răspunde DOAR pe baza
informaţiilor de mai sus, chiar
dacă acestea par incorecte sau
diferite de cunoştinţele tale
generale.
Întrebare: Care este capitala
României?
Te rog să:
1. Scrii o explicaţie în limba
română (1-2 paragrafe).
2. La final, răspunzi în format
JSON.
IMPORTANT: ÎNTÂI scrie explicaţia,
APOI blocul JSON.
Format JSON: {"answer": "răspunsul
tău"}
```

English (translation)

```
You have the following database of
information available:
 • Capital of Romania: Sibiu
 • Longest river: Danube
 • Highest peak: Moldoveanu
 • Population: 19 million
WARNING: Answer ONLY based on the
information above, even if it
seems incorrect or different from
your general knowledge.
Question: What is the capital of
Romania?
Please:
1. Write an explanation in
Romanian (1-2 paragraphs).
2. At the end, answer in JSON
format.
IMPORTANT: FIRST write the
explanation, THEN the JSON block.
JSON format: {"answer": "your
answer"}
```

Figure S2. Example Fact World prompt with "misbelief trap" (bilingual). The context states Romania's capital is Sibiu (incorrect—the actual capital is Bucharest). A well-instructed model should answer "Sibiu" based on context, subordinating parametric knowledge.

Table S2. Comparison of Original vs. Romanian-Finetuned Models. All three model families show consistent degradation after Romanian finetuning, with Understanding (U) and JSON success rates most affected.

Base Model	Variant	U	JSON%	Final
Llama-3.1-8B	Original	0.774	95%	85.6%
	RoLlama3.1-8B	0.421	44%	65.8%
	Δ *Degradation*	*−0.353*	*−51pp*	*−19.8pp*
Gemma-2-9B	Original	0.823	99%	82.2%
	RoGemma2-9B	0.546	70%	71.3%
	Δ *Degradation*	*−0.277*	*−29pp*	*−10.9pp*
Gemma-7B	Original	0.662	89%	72.6%
	RoGemma-7B	0.318	36%	58.6%
	Δ *Degradation*	*−0.344*	*−53pp*	*−14.0pp*

pp = percentage points

Romanian (original)

```
Ai un calendar pentru zilele:
Luni, Marţi, Miercuri.
Fiecare zi are două intervale:
dimineaţă, după-amiază.
Următoarele programări trebuie
organizate:
 • Control medical (prioritate:
înaltă, programat: Luni dimineaţă)
 • Şedinţă de proiect (prioritate:
medie, programat: Marţi
după-amiază)
 • Antrenament sportiv (prioritate:
scăzută, programat: Miercuri
dimineaţă)
 • Întâlnire cu clientul
(prioritate: înaltă, programat:
Luni după-amiază)
Respectă următoarele cerinţe:
 - Maxim 2 programări pe zi.
 - Trebuie să păstrezi toate
programările cu prioritate înaltă.
IMPORTANT: ÎNTÂI scrie explicaţia,
APOI blocul JSON.
Format JSON: {"Luni_dimineaţă":
"...", ...}
```

English (translation)

```
You have a calendar for the days:
Monday, Tuesday, Wednesday.
Each day has two slots: morning,
afternoon.
The following appointments must be
organized:
 • Medical checkup (priority: high,
scheduled: Monday morning)
 • Project meeting (priority:
medium, scheduled: Tuesday
afternoon)
 • Sports training (priority: low,
scheduled: Wednesday morning)
 • Client meeting (priority: high,
scheduled: Monday afternoon)
Respect the following
requirements:
 - Maximum 2 appointments per day.
 - You must keep all high-priority
appointments.
IMPORTANT: FIRST write the
explanation, THEN the JSON block.
JSON format: {"Luni_dimineaţă":
"...", ...}
```

Figure S3. Example Schedule World prompt (bilingual). The model must organize appointments into calendar slots while respecting priority constraints. Note that the JSON keys use Romanian day/slot names, testing diacritic handling in structured output.

Romanian (original)

```
Trebuie să planifici meniurile
pentru 2 zile.
Preparate disponibile:
Mic dejun:
 • Ouă jumări cu roşii (vegetarian,
250 kcal)
 • Mămăligă cu brânză (vegetarian,
conţine lactoză, 400 kcal)
 • Smoothie verde cu spanac (vegan,
180 kcal)
Prânz:
 • Ciorbă de legume (vegan, 200
kcal)
 • Sarmale în foi de viţă (450
kcal)
 • Salată grecească (vegetarian,
250 kcal)
Cină:
 • Supă cremă de ciuperci
(vegetarian, 220 kcal)
 • Orez cu legume (vegan, 300
kcal)
Cerinţe:
 – Toate preparatele trebuie să
fie vegetariene.
 – Totalul caloriilor pe zi: max
1500 kcal.
IMPORTANT: ÎNTÂI explicaţia, APOI
blocul JSON.
```

English (translation)

```
You must plan menus for 2 days.
Available dishes:
Breakfast:
 • Scrambled eggs with tomatoes
(vegetarian, 250 kcal)
 • Polenta with cheese (vegetarian,
contains lactose, 400 kcal)
 • Green spinach smoothie (vegan,
180 kcal)
Lunch:
 • Vegetable soup (vegan, 200
kcal)
 • Stuffed cabbage rolls (450
kcal)
 • Greek salad (vegetarian, 250
kcal)
Dinner:
 • Cream of mushroom soup
(vegetarian, 220 kcal)
 • Rice with vegetables (vegan,
300 kcal)
Requirements:
 – All dishes must be vegetarian.
 – Total daily calories: max 1500
kcal.
IMPORTANT: FIRST explanation, THEN
JSON block.
```

Figure S4. Example Recipe World prompt (bilingual). The model must plan meals satisfying dietary restrictions (vegetarian) and caloric limits. Note that “Sarmale” (cabbage rolls) contains meat and must be excluded despite being a traditional Romanian dish.
